# A village in a dish model system for population-scale hiPSC studies

**DOI:** 10.1038/s41467-023-38704-1

**Published:** 2023-06-09

**Authors:** Drew R. Neavin, Angela M. Steinmann, Nona Farbehi, Han Sheng Chiu, Maciej S. Daniszewski, Himanshi Arora, Yasmin Bermudez, Cátia Moutinho, Chia-Ling Chan, Monique Bax, Mubarika Tyebally, Vikkitharan Gnanasambandapillai, Chuan E. Lam, Uyen Nguyen, Damián Hernández, Grace E. Lidgerwood, Robert M. Graham, Alex W. Hewitt, Alice Pébay, Nathan J. Palpant, Joseph E. Powell

**Affiliations:** 1 Garvan-Weizmann Centre for Cellular Genomics, Garvan Institute of Medical Research, Darlinghurst, 2010 Sydney Australia; 2 Graduate School of Biomedical Engineering, University of New South Wales, Kensington, 2033 Sydney Australia; 3 Institute for Molecular Bioscience, University of Queensland, Brisbane, Australia; 4 Department of Anatomy and Physiology, the University of Melbourne, Melbourne, Australia; 5 Victor Chang Cardiac Research Institute, Darlinghurst, NSW Australia; 6 UNSW Medicine & Health, UNSW Sydney, Kensington, NSW Australia; 7 St Vincent’s Hospital, Darlinghurst, 2010 NSW Australia; 8 Centre for Eye Research Australia, Royal Victorian Eye and Ear Hospital, University of Melbourne, Melbourne, Australia; 9 School of Medicine, Menzies Institute for Medical Research, University of Tasmania, Hobart, Australia; 10 Department of Surgery, Royal Melbourne Hospital, Anatomy and Neuroscience, the University of Melbourne, Melbourne, Australia; 11 UNSW Cellular Genomics Futures Institute, School of Medical Sciences, University of New South Wales, 2052 Sydney, Australia

**Keywords:** RNA sequencing, Gene expression, Stem-cell biotechnology, Induced pluripotent stem cells

## Abstract

The mechanisms by which DNA alleles contribute to disease risk, drug response, and other human phenotypes are highly context-specific, varying across cell types and different conditions. Human induced pluripotent stem cells are uniquely suited to study these context-dependent effects but cell lines from hundreds or thousands of individuals are required. Village cultures, where multiple induced pluripotent stem lines are cultured and differentiated in a single dish, provide an elegant solution for scaling induced pluripotent stem experiments to the necessary sample sizes required for population-scale studies. Here, we show the utility of village models, demonstrating how cells can be assigned to an induced pluripotent stem line using single-cell sequencing and illustrating that the genetic, epigenetic or induced pluripotent stem line-specific effects explain a large percentage of gene expression variation for many genes. We demonstrate that village methods can effectively detect induced pluripotent stem line-specific effects, including sensitive dynamics of cell states.

## Abstract (web summary)

Village cultures, where multiple stem cell lines are cultured in a single dish, provide an elegant solution for population-scale studies. Here, authors show the utility of village models – showing that expression heterogeneity is largely a result of line-specific effects and not village cultures.

## Introduction

Using human induced pluripotent stem cells (hiPSCs) and their derivatives to study complex human traits such as diseases and drug responses is becoming a new research frontier through the intersection with population genetic approaches^1–4^. hiPSCs are karyotypically normal, self-renewable cells that are generated by reprogramming human somatic cells. They can differentiate into virtually any cell type in the human body^5^, providing a model system to study human cell types in vitro. Recent work has demonstrated that hiPSCs are a powerful system for investigating large-scale inter-individual variation and context-dependent effects that would be challenging to recreate in vivo. Here, we consider context-dependent effects genetic relationships with phenotypes only detectable under specific conditions. For example, some expression quantitative trait loci (eQTLs) are only detected in specific tissues^6^, cell types^2,3,7^, cell states^8^, or following drug^9^ or chemokine^10^ exposure. While hiPSCs are a powerful model system to interrogate these context-specific effects, large-scale hiPSC culture is expensive and time-consuming, creating challenges for studies that require hundreds to thousands of donor lines. Previous studies have relied on bulk RNA sequencing (RNA-seq) to assess gene expression, which has two major limitations. First, it effectively averages the heterogeneous expression of different cell types into a single measure. Secondly, the types of experimental design used in bulk RNA-seq require each hiPSC line to be cultured, extracted and processed independently—creating inherent correlations between line and batch effects.

To mitigate these limitations, recent studies have applied a village approach to culture hiPSCs—where multiple unrelated lines are cultured and differentiated in a single dish^2,8,11^. One of these studies paired flow sorting of survival motor neuron protein levels with whole genome sequencing. The proportion of each hiPSC line in each survival motor neuron flow-sorted group of cells were estimated with computational methods. The study design provided statistical power to detect survival motor neuron protein quantitative trait loci—genetic variants associated with protein expression levels of cell lines. While this approach is effective, it is not easily scalable for high-throughput assays of molecular phenotypes. Other studies have applied village culture methods with single-cell RNA-sequencing (scRNA-seq). Each cell is assigned to a line in the pool in tusing demultiplexing methods^12–15^. However, the impact on molecular phenotypes due to multiple cell lines in a single village culture has not been assessed.

This is a challenge, as it needs to be clarified whether cell signaling in villages will influence the transcriptional profiles of each independent hiPSC line. If such effects were to exist, they would likely lead to biases in the identification of both eQTLs and context-dependent effects due to the creation of ‘artificial’ correlations in the phenotypes between donor lines. Here, we develop village culture systems and demonstrate their efficacy for population-scale stem cell studies. We investigate how growth rates impact the proportions of cells from each line in the village and whether cell signaling alters the transcriptional profiles of individual cells in village culture conditions. We evaluated these properties across multiple independent laboratories.

We show that the inter-line gene expression is unaffected by culturing lines in a village or between sites. In other words, the genetic, epigenetic or line-dependent effects that underlie gene expression variation between different lines are consistent when the cells are cultured separately or in a village. Furthermore, we demonstrate that line-specific effects that change across a cell-state pseudotime (dynamic effects) can be reproducibly detected, further supporting the use of village culture systems for large-scale studies with hiPSCs. Our results demonstrate that the village model can be effectively used to detect eQTLs and other cell line-specific effects, and we provide important details that will allow these approaches to be easily implemented in different laboratories.

## Results

### Experimental and analytical framework

**Fig. 1:**
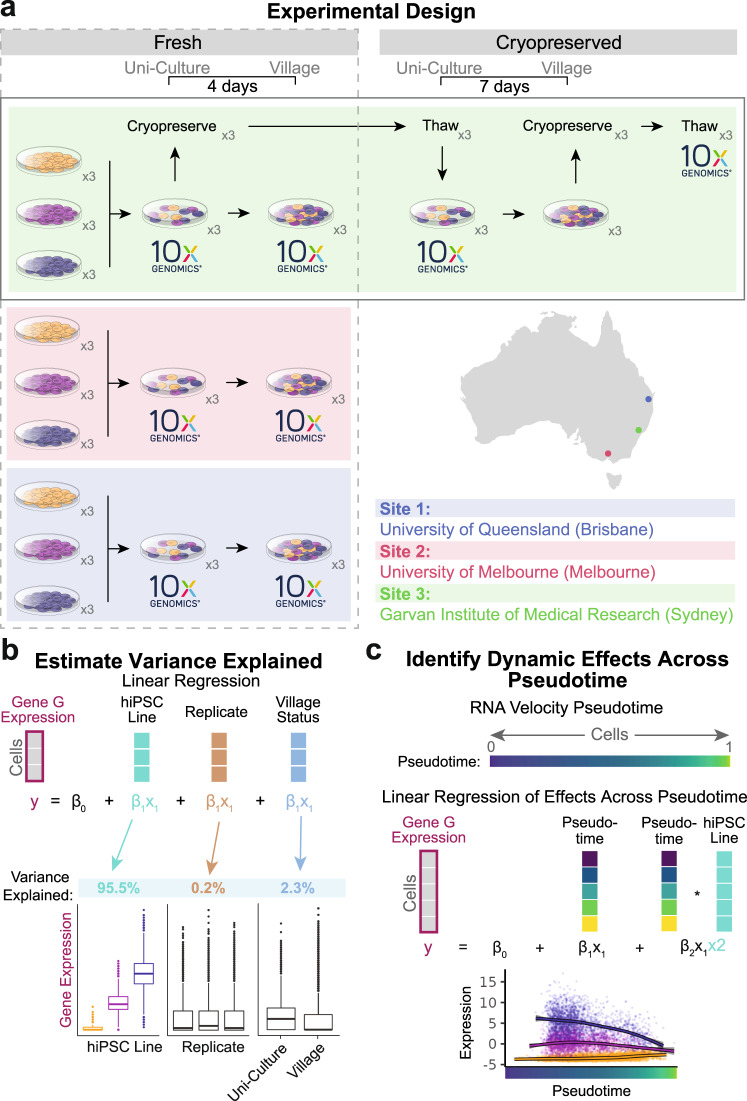
Experimental design and analytical approaches. **a** The experimental design to test for the impact of village culture conditions on individual hiPSC lines using scRNA-seq. Phase I compares the impact of the village culture system using fresh samples collected at each site. Phase II investigates whether cryopreservation of village samples impacts individual hiPSC lines. Phase III utilizes all samples to investigate dynamic effects of the hiPSC lines across pseudotime. Each phase utilizes expression matrices that are separated by condition (site for phase I, cryopreservation status for phase II and pseudotime for phase III) as well as covariate matrices for each condition that contain the hiPSC line, replicate and village status. **b** A linear mixed model is used to estimate the variance of expression for each gene that is explained by each covariate. Those estimates are calculated for each condition in each Phase of the experimental design and used for downstream analyses. **c** The pseudotime is estimated for the cells from all samples and used in a linear mixed model to identify genetic effects that are dynamic across pseudotime. ×3: samples in triplicate; 10x Genomics: 10x Genomics scRNA-seq capture. All figure parts are representative.

We implemented a multi-phased experimental design that enabled the interrogation of village culture conditions while also comparing inter-laboratory and cryopreservation effects using scRNA-seq (Fig. 1a).

To compare the effects of village culture conditions in different laboratories, Phase I involved the generation of data from three independent sites—the University of Queensland (Brisbane, Australia; Site 1), the University of Melbourne (Melbourne, Australia; Site 2) and the Garvan Institute of Medical Research (Sydney, Australia; Site 3). The same lines from the same passage (FSA0006, MBE1006 and TOB0421), the same protocols and the same reagents (from the identical batches) were used at each site with one exception—lines were plated at a lower density at Site 3 (~1/10 the plating density used at Sites 1 and 2). Villages were generated using equal proportions of each uni-cultured line (cultured in separate dishes) and maintained for four days before single cell capture. For the scRNA-seq capture of uni- and village cultures in Phase I, cells were detached and dissociated simultaneously at each site and placed on ice. Samples from Sites 1 and 2 were transported to Site 3, where the samples were processed and captured together (Fig. 1a; see “Methods” for additional details), mitigating capture batch effects. Phase II investigated the potential impact of cryopreservation on inter-line effects in the village culture system (maintained for seven days), which was performed at Site 3 (Fig. 1a). Finally, Phase III used data from all cells to investigate dynamic hiPSC line effects across cell-state pseudotime (inter-line effects that change over pseudotime; Fig. 1a).

In the analysis of the scRNA-seq data, we estimated the variance explained by hiPSC line, replicate, site, cryopreservation and village status and the interaction of each of those covariates using a linear mixed model (Fig. 1b). Finally, for Phase III, the gene expression variance explained by the lines was tested for dynamic effects across pseudotime with a linear mixed model to identify inter-line effects that change over pseudotime (Fig. 1c).

### Impact of village culture system

**Fig. 2:**
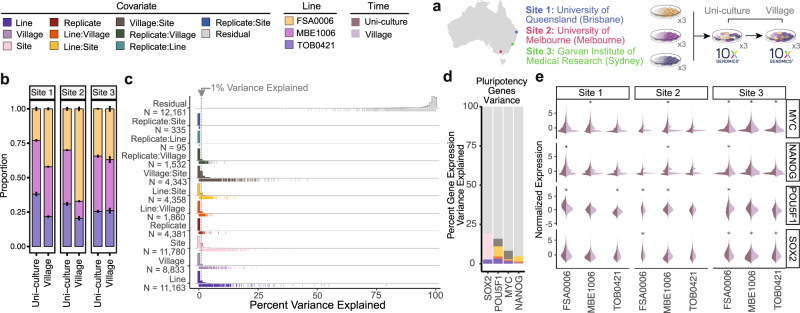
Impact of village culturing system. **a** Experimental design to test the impact of village culturing systems on hiPSC transcriptional profiles. The same experiment was carried out at each of the three different sites in triplicate. **b** The proportions of each of the three hiPSC lines at each of the three different sites from uni- and village cultures. Error bars show the standard error of the triplicates around the mean. **c** Histograms of the variance of gene expression that is explained by the covariates and the interaction of the covariates measured. The lines below the histogram each represent a gene for that covariate. **d** The variance of important stem cell markers explained by the covariates. **e** Important stem cell marker expression for each hiPSC line at each Site. Differential expression was calculated with logistic regression and significance was corrected for multiple comparisons. hiPSC: human induced pluripotent stem cell; *adjusted *P*-value < 0.05 for differential expression.

To investigate the potential impacts of village culture conditions on individual lines, we collected samples of the lines cultured separately and, after four days, cultured in a village at the three independent sites as previously described (Figs. 1a and 2a; see “Methods” for additional details). In addition, since some experimental designs require cryopreserving cells at different time points, we also investigated village effects by comparing fresh and cryopreserved uni-culture and village samples (Figs. 1a, 2a and Supplementary Fig. 1a; see “Methods” for additional details).

### Proportions of hiPSC lines following village culturing

Village culture systems provide advantages over uni-culture systems, provisional that the majority of the hiPSC lines can be maintained in culture. Therefore, we first compared the proportion of each line when they were first pooled (uni-culture) to the proportions following culture in a village. After demultiplexing the samples (see Methods), we found that all lines were present in all samples, albeit at different proportions in the village than the uni-culture samples at Sites 1 and 2 but not 3. At Sites 1 and 2, the village samples had a larger proportion of FSA0006 and a smaller proportion of MBE1006 and TOB0421 (Fig. 2b and Supplementary Fig. 2a–b). These results are consistent considering the reduced plating density at Site 3. Furthermore, the cryopreserved samples from Site 3 demonstrated consistency with the fresh samples from Sites 1 and 2—differences in the line proportions between the cryopreserved uni- and village samples—especially FSA0006 as indicated by *scCODA* (Supplementary Fig. 1b–d).

We tested whether variation in hiPSC line growth rates could contribute to variation in the proportion of each line in the village and demonstrated that FSA0006 had almost double the growth rate of the other two lines (Supplementary Fig. 3). The different proportions of each line in the village suggest that growth rate is important when designing village experiments for long-term cell culture.

### Transcriptional profiles are unaffected by village culturing

To evaluate whether cell signaling of companion lines in a village alters the transcriptional profiles of other lines, we calculated the pairwise correlation of the transcriptome profiles of each sample and compared the distributions for each covariate. The highest correlations were observed between replicates (median rho = 0.99) followed by Village (median rho = 0.98), line (median rho = 0.98) and Site (median rho = 0.97; Supplementary Fig. 2c). A similar pattern of correlations was observed for the cryopreserved samples with the most similar expression profiles between replicates (median rho =  0.99), followed by Cryopreservation (median rho = 0.98), line (median rho = 0.97) and Village (median rho = 0.97; Supplementary Fig. 1e). This demonstrates that the transcriptional profiles of each line are more different than those between uni-culture and village samples for the same line (Supplementary Fig. 2c).

To evaluate the effect of culturing hiPSC lines in a village on each gene, we utilised a linear mixed model framework to estimate the percentage of the variance of gene expression that was explained by the line, replicate, village, site, cryopreservation or interaction of each variable in the Fresh (Fig. 2c and Supplementary Data 1) and Cryopreserved samples (Supplementary Figs. 1f and 2; see “Methods” for details). High estimates of the variance explained by the interaction of two covariates indicate that they impact gene expression variation more than the addition of the two variables alone. For example, if the interaction between the line and the village status is significant for a given gene, it would suggest that the village status is altering the variance of the hiPSC alone or vice versa—presumably by having a greater impact on a specific line.

We observed that, on average, the hiPSC line explains 0.71% of gene expression variance (inter-line variation), the village status explains 0.22%, and the interaction of line and Village covariates (Line:Village) only explains 0.32% of the variation in the fresh samples (Fig. 2c and Supplementary Data 1). However, in the cryopreserved samples, the average variance explained was similar between the line (1.13%), the Village (2.19%) and the interaction of the Line and Village (1.13%). When considering just variance that explained at least 1% of gene expression variation, more genes were influenced by the Line (Fresh: 1,824; Cryopreserved: 2,401) than the interaction of the Line and the Village (Fresh: 113; Cryopreserved: 980, Fig. 2c, Supplementary Fig. 1f and Supplementary Data 1 and 2). This indicates that while the village impacts the expression of some genes, the differences in gene expression between lines are detectable and, on average, have a larger effect.

Cryopreservation followed a similar pattern, with the interaction between the line and Cryopreservation explaining 0.63% of the gene expression variance on average, while the line alone explained 1.13%. Further, only 113 genes had >1% of their variance explained by the interaction of the line and Cryopreservation as opposed to 2,401 by the line alone (Supplementary Fig. 1f and Supplementary Data 2). This further indicates that cryopreservation has a limited impact on the gene expression variation conferred by the hiPSC lines.

Since two of the hiPSC lines were generated from male donors and one from a female donor, the Y chromosome gene expression variance can be used as a positive control for line effects. Indeed, we observed that lines are the largest contributor to Y chromosome gene variance (Supplementary Figs. 2d, 4a, and Supplementary Data 3). These results suggest that culturing lines in a village system do not significantly alter the transcriptome of each hiPSC line.

### hiPSC line effects of pluripotency genes are not impacted by villages

It is important to understand the impact (if any) that the village culture system could have on gene expression denoting a pluripotent state. We identified that, on average, only a small percentage of the total variance of pluripotency gene expression was explained by the village status for common stem cell markers such as *MYC*, *NANOG*, *SOX2* and *POU5F1*^5^ (Fig. 2d, e, Supplementary Figs. 2e and 4b–d). Furthermore, while some of the pluripotent markers demonstrated significant differences in expression between the village and uni-culture samples, most effect sizes are small (Fig. 2e and Supplementary Fig. 2f).

These results suggest that culturing hiPSCs in a village and cryopreservation of villages do not significantly alter the proportions of each line or their unique transcriptional profiles and suggest that village systems are appropriate for population genomic studies.

### eQTL allelic effects are not altered by village culture

**Fig. 3:**
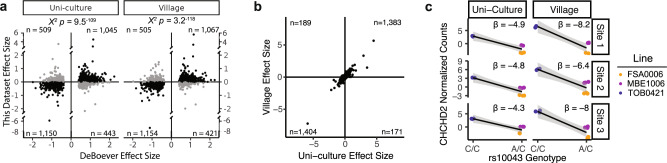
eQTL detection consistent in Uni-culture and Village samples. **a** Replication of eQTLs previously described by DeBoever et al. Significance detected with a two-sided Chi squared test. **b** Two-sided Spearman rank correlation between the effect sizes of previously reported eQTLs in the Uni-culture (*x*-axis) and Village (*y*-axis) samples. **c** The previously reported eQTL for *CHCHD2* demonstrates a strong and consistent effect across different Sites and the Village status. The gray band around the line indicates the standard error.

We next investigated whether we could detect previously reported eQTLs^16^ with our data. Indeed, the majority of eQTLs previously identified by DeBoever et al. that had at least two genotypes in this dataset demonstrated consistent effects in both the uni-culture (Fresh: 69.7%, Cryopreserved: 63.9%) and village samples (Fresh: 70.6%, Cryopreserved: 66.8%; Fig. 3a and Supplementary Fig. 5a). Furthermore, 88.6% of loci had a consistent direction of allelic effects between the village and uni-culture samples (Figs. 3b and S5b). An example, the single nucleotide polymorphism (SNP) rs10043 was consistently associated with *CHCHD2* expression in both uni-culture and village samples for fresh (−4.3 < β < −8.2; Fig. 3c) and cryopreserved (−4.3 < β < −11.7; Supplementary Fig. 5c). As reported previously, the reference C allele was associated with higher expression than the alternate A allele. These data demonstrate that line-specific effects such as eQTLs can be consistently detected using village culture systems. These results support the conclusion that culturing lines in a village model is unlikely to impact the ability to identify line-dependent transcriptional effects such as eQTLs.

### Dynamic effects of hiPSC line across cell state pseudotime

Our results conclude that culturing cells in village conditions does not significantly alter inter-line variation in gene expression across cells. However, our results until this point only consider the potential variation in cell states. Therefore, we next sought to determine the variance in gene expression explained in different cell states—for example, differences in pluripotent potential or cells spontaneously differentiating.

**Fig. 4:**
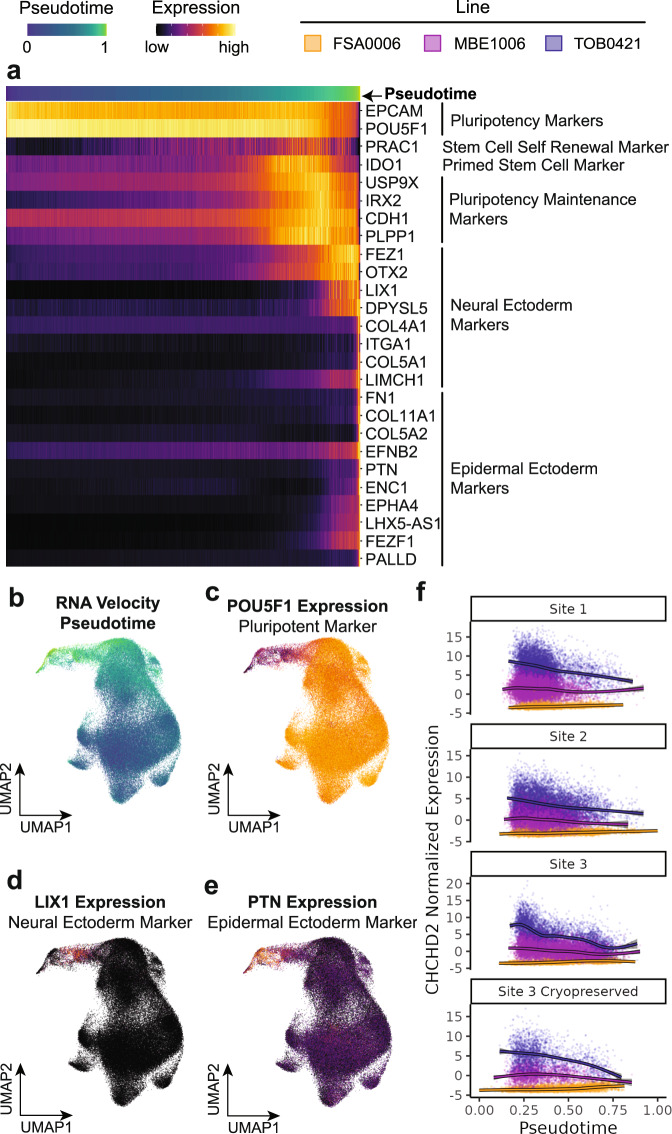
Dynamic variance explained across stem cell pseudotime. **a** Pseudotime appeared to define cell pluripotency as evidenced by pluripotency markers and ectodermal markers expressed higher pseudotime corresponding to spontaneously differentiated cells. **b** The pseudotime projected onto the UMAP of all cells. **c**–**e** Markers representative of pseudotime progression: pluripotency (*POU5F1*; **c**), Neural Ectoderm (*LIX1*, **d**) and Epidermal Ectoderm (*PTN*; **e**). **f** The dynamic interaction of pseudotime with the *CHCHD2* gene—hiPSC line effects are larger at smaller pseudotime values (pluripotent cells) and smaller at larger pseudotime values (spontaneously differentiated cells). The band around the lines represents the standard error. hiPSC: human induced pluripotent stem cell.

Using RNA velocity, we first positioned cells based on their estimated pseudotime trajectory (Fig. 1d and Supplementary Fig. 6a). As expected, we observed that the pseudotime landscape was strongly defined by the cells’ pluripotency (Fig. 4a, b and Supplementary Fig. 6b), with lower pseudotime corresponding to pluripotent markers and higher pseudotime coinciding with markers of spontaneous differentiation into the ectoderm lineages (Fig. 4a–e). Next, we interrogated the contributions of pseudotime to gene expression variation using a linear mixed model approach similar to that used in Phases I and II. Again, as expected, pseudotime explains a large percentage of gene expression variation for many genes (on average 3.8%), with many genes where >1% of the variance is explained by Pseudotime (1924; Fig. S6b).

### hiPSC line effect is dynamic across pseudotime

We identified 1965 significant dynamic line effects—inter-line variation that consistently decreases or increases across pseudotime (FDR < 0.05, Supplementary Fig. 6c and Supplementary Data 4). Using *CHCHD2* as an example again, we observe a strong dynamic effect across pseudotime with larger differences in expression between the lines in more pluripotent cells (Fig. 4c).

### Large hiPSC villages demonstrate consistent results

**Fig. 5:**
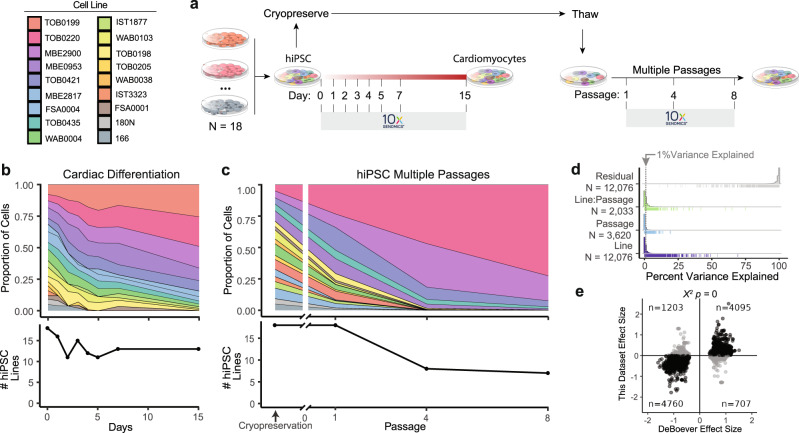
Large hiPSC village differentiation and maintenance. **a** Experimental design for testing a large village of 18 unrelated hiPSC lines including the cell numbers and transcriptional profiles during cardiomyocyte differentiation and hiPSC village maintenance. **b** Proportion of cells from each hiPSC line during cardiomyocyte differentiation (15-day protocol). **c** Proportion of cells from each hiPSC line during hiPSC village culture maintenance. **d** Proportion of gene expression variance during hiPSC village maintenance explained by the covariates measured in this dataset—Line and Passage. Each line in the rug plot below the histogram shows the observation of a gene for that covariate. **e** Replication of eQTLs previously described by DeBoever et al. with the 18-line iPSC village with 83% of eQTLs in concordant directions. Significance identified with two-sided Chi-squared test.

Next, we examined the effects of a village containing 18 iPSC lines, which were differentiated into cardiomyocytes and cultured for multiple passages and in two separate experiments (Fig. 5a). The village of 18 lines was generated when initiating the cardiomyocyte differentiation, and the proportion of cells was assessed at eight-time points during cardiomyocyte differentiation. The village of 18 iPSC lines was also cryopreserved at Day 0 and thawed later to evaluate the effect of culturing villages over multiple passages—assayed at passages one, four and eight (Fig. 5a and Supplementary Data 6–7).

Five lines were lost in the village over the 15-day cardiomyocyte differentiation, with the largest loss of lines occurring in the first two days. Ten lines were lost by passage eight of the village—most of which had already been lost by passage four. Consistent with previous results, the growth rates of the lines were correlated with the proportion of each iPSC line in the village during cardiomyocyte differentiation (Supplementary Fig. 7a) and following multiple passages (Supplementary Fig. 7b). anticipated, the correlation was insignificant at Day 0 of cardiomyocyte differentiation as the growth rates are unlikely to contribute to human variation in cell counting and pooling, resulting in each line representing 3–9% of the village. These results support the conclusion that growth rates are important to village sustainability and must be carefully assessed for long-term village cultures.

The variation of proportions of cells in each single-cell capture is similar to previous pooled single-cell experiments (Supplementary Fig. 8), and the village experiments are similar or smaller than previous village experiments (Supplementary Fig. 8). Further, the variation in cell numbers observed for the cardiomyocyte differentiation village was smaller than that for the pooled cardiomyocyte differentiation.

We next interrogated the impact of the covariates on the variation in gene expression. Importantly, the iPSC line explains a significant percentage of the variation in gene expression for more genes than the passage or the interaction of the line and the passage (Fig. 5d and Supplementary Data 5). Finally, we were able to replicate 83% of eQTLs that could be measured in the 18-line village, demonstrating that the village impact does not alter the ability to estimate unique gene expression identities.

Our results demonstrate that village culture methods can be effectively used for population genomic studies with lines. However, care must be taken with the lines selected for a village to ensure that a single line does not dominate the village. We have also demonstrated that cryopreservation does not alter the transcriptional profiles or line proportions, and even sensitive dynamic effects can be reproduced with village systems.

## Discussion

Advances in human genetics, stem cell biology, and single-cell technologies have led to the convergence of these research domains that allow better evaluation of the complexity of human genetic regulation. Population genomic studies using hiPSCs and hiPSC-derived cells have steadily increased in sample size and frequency as more lines have become available and methods for culturing hiPSC-derived cells have developed. However, maintaining hiPSCs and hiPSC-derived cells is still expensive and time-consuming, compromising the ability to consistently apply large-scale population genomic studies.

To date, the majority of population genetics hiPSC studies have been conducted using bulk sequencing methods—which means that the transcriptomes of all the cells in a sample are combined and assayed together as one. These studies have led to significant new knowledge of the role of genetic variation on gene regulation. Still, bulk sequencing approaches effectively remove sample heterogeneity, which is important for cell type-specific and context-specific effects. In contrast, single-cell technologies provide a powerful solution for this challenge, as individual cells are assayed separately, and context-specific effects can be interrogated. Indeed, a few recent studies have demonstrated that single-cell and deconvolution methods applied to hiPSCs and hiPSC-derived cells can be used to detect pQTLs^11^, eQTLs and context-specific effects^2–4,8^.

Studies that have used village culture systems coupled with scRNA-seq have employed computational demultiplexing approaches to obtain RNA measures for each cell from each line effectivly^2,8^. These village culture systems address an additional limitation of bulk RNA-seq methods—batch effects are wholly confounded with line effects since each line has historically been cultured in a separate cell culture dish. Previous village hiPSC-derived studies have identified context-dependent eQTLs but have not assessed the potential impact of cell signaling in the village culture system and whether it alters the transcriptional profiles of companion lines. They detected fewer eQTLs per cell type than anticipated for the sample sizes. This could have been partially due to the introduction of non-genetic variance resulting from the village model^2,8^. Therefore, to confidently use village models for future population-scale experiments, there was a critical need to thoroughly assess whether using a village culture system would alter the transcriptional profiles of the cells from each line. Our experimental procedure addressed that question and provided a roadmap for village culture experimental design.

Our results demonstrate that, while it is important to consider line growth rates, village experimental culture systems do not alter the unique transcriptional profiles of each line and are, therefore, an applicable system for population-scale experiments. Importantly, we could detect multiple lines in all samples and consistently identify line-dependent variation. Transcriptional profiles were consistent in villages and at different sites, demonstrating that village experiments could theoretically be conducted across multiple sites. In addition, cryopreservation had a minimal detectable effect on transcriptional profiles that altered the line effects. Therefore, line-dependent studies like QTL studies can effectively be carried out using village culture designs.

Importantly, we replicated previous eQTLs identified by DeBoever et al. with bulk RNA-seq, including the important neuralectordermal stem cell priming gene *CHCHD2*^17^. Intriguingly, *CHCHD2* showed dynamic effects across pseudotime, with the largest difference in line expression observed in the most pluripotent cells where *CHCHD2* has the most important priming role—possibly identifying a genetic and expression marker that can be used to classify lines more primed for neuroectoderm differentiations.

Further, the use of the 18-line village for cardiomyocyte differentiation and maintenance for eight passages confirmed that lines with higher growth rates are likely to compose a larger proportion of the village than lines with lower growth rates. This was especially noticeable following multiple passages and is consistent with the village that contained three lines and previous findings^11^. Regardless, village culture systems provide key advantages by increasing the number of lines that can be concurrently cultured and assayed—significantly increasing scale.

It is important to note that, like any model system, hiPSCs have limitations to consider including that hiPSC-derived cells are forced differentiations that, historically, reach a plateau at fetal maturity. In addition, hiPSC differentiations are removed from the whole-organ or whole-body context. There is ongoing research in these spaces to help diminish these limitations. However, hiPSC-derived models provide unfettered access to cell types that can be difficult to obtain from living human donors and allow specific modulation of the cellular environments—enabling the identification of context-specific effects.

While hiPSC differentiations were not the main focus of this study, we showed that multiple lines could be maintained in a village during cardiomyocyte differentiation. Some previous village studies have been carried out for NPCs^18^ and dopaminergic neuron^2^ differentiations. Naturally, post-mitotic differentiations, such as neuronal differentiations are likely to be the most successful for early village studies since cells stop expanding, and it may be easier to manage line proportions. Nevertheless, our results demonstrate that pooling lines with similar growth rates will significantly help maintain multiple lines in a single village.

The advantages gained from using single-cell data to obtain purer cell subtypes and leveraging village culture systems to increase throughput are extensive. In our opinion, these advantages significantly outweigh the limitations of hiPSCs. Village systems—paired with single-cell technologies—promise to revolutionise the field of population genomics of gene regulation.

## Methods

### Ethics

The research carried out in this study was conducted in accordance with the Declaration of Helsinki and approved by the Human Research Ethics committees of the University of Melbourne (1545394), the Garvan Institute of Medical Research (ETH01307) and the University of Queensland (2015001434). All patients gave written informed consent to collect and use their biological data for this research.

### Three-line village

#### hiPSC cell culture

1 mL aliquots of each of the hiPSC lines FSA0006, MBE1006 and TOB0421^19^ (passage 18; Supplementary Data 3) were thawed at each site on the same day and plated in StemFlex^TM^ media (Life Technologies; Catalog Number: A3349401; Lot Number: 2093181) complete with StemFlex^TM^ Supplement (Life Technologies, Catalog Number: A33492-01; Lot Number: 2090179) with Rock inhibitor Y-27632 (10 µM final concentration; Stem Cell Technologies; Catalog Number: 72304) on Costar non-treated six well polystyrene plates (Catalog Number: 3736; Lot Number: 30417038) coated with Vitronectin XF (Stem Cell Technologies; Catalog Number: 07180; Lot Number: 18B87584) diluted in CellAdhere Buffer (Stem Cell Technologies, Catalog Number: 07183; Lot Number: 18M979058). Cells were subcultured once a week with ReLeSR for two weeks (Stem Cell Technologies; Catalog Number: 05872) before equal numbers of the hiPSC lines were combined and plated together. The same lot number of all reagents were used between the three locations (the University of Queensland in Brisbane, the University of Melbourne in Melbourne and the Garvan Institute of Medical Research in Sydney). hiPSC villages were cryopreserved in 1 mL with CryoStor® CS10 (STEMCELL Technologies; Catalog Number: 100–1061) per manufacturer instructions.

#### scRNA-seq capture

Totalseq-A antibodies (Biolegend; Catalog Numbers: 394607, 394613, 394601, 394609, 394615, 394603, 394605, 394611) were used to hashtag hiPSC pools from each different location (the University of Queensland, Garvan Institute of Medical Research and University of Melbourne) before combining and super-loading onto the 10x Genomics Chromium Controller (10x Genomics) to capture single cells. Briefly, 1 × 10^6^ cells from each replicate at each site were centrifuged at 300 × *g* for three minutes and the supernatant was discarded. The cell pellets were resuspended in 100 µL cold Fluorescence-Activated Cell Sorting (FACS) buffer (phosphate buffered saline [PBS] with two percent fetal bovine serum [FBS]). Then, 2 µL of Totalseq-A hashing antibody was added and the cells were gently pipetted to mix before incubating for 20 min on ice. Cells were then washed twice with FACS buffer by centrifuging at 300 × *g* for 5 min, discarding the supernatant and resuspending the cell pellet in 100 µL cold FACS buffer. Cells were briefly stained with 4′,6-diamidino-2-phenylindole (DAPI) before using flow cytometry (BD FACS Aria, 100 µm nozzle, four-way purity mode, temperature controlled) to sort and capture live single cells. Cell pools contained one sample from each site (Supplementary Fig. 9). Trypan blue was then used to assess the pool viability (>75% viable). Approximately 32,000 cells were loaded onto the Chromium Single Cell Chip B (10x Genomics; Catalog Number: PN-1000073) to capture 20,000 single cells with the Gel Bead Kit V3.0 (10x Genomics; Catalog Number: PN-1000076).

GEM generation, barcoding, cDNA amplification, and library construction were performed according to the 10x Genomics Chromium User Guide (CG000183). Libraries were prepared with the Single Cell 3’ V3.0 Library and Gel Bead Kit (10x Genomics; Catalog Number: PN-1000077 and PN-1000078).

#### Growth Rate Estimation

hiPSC lines FSA0006, MBE1006 and TOB0421 were each plated in two six-well plates on day 0 in StemFlex^TM^ media complete with StemFlex^TM^ Supplement with Rock inhibitor Y-27632 on Costar non-treated six-well polystyrene plates coated with Vitronectin XF. Media was changed on day 2 and then cells from three wells were detached and counted on days 4, 5, 6, and 7 each. The resulting cell counts were used to estimate growth rates with *ratrack*^20^.

### Eighteen-line village

#### hiPSC cell culture

1 mL aliquots of each of the hiPSC lines (Supplementary Data 3)^19^ were thawed and plated in mTeSR^TM^ Plus media complete with mTeSR^TM^ Plus Supplement (Life Technologies, Catalog Number: 100–0276) with Rock inhibitor Y-27632 (10 µM final concentration; Stem Cell Technologies; Catalog Number: 72304) on Costar TC-treated six-well plates (Corning; Catalog Number: 3516) coated with Matrigel® hESC-Qualified Matrix (Corning; Catalog Number: 354277) diluted in Dulbecco’s Modified Eagle Medium/F-12 (ThermoFisher Scientific, Catalog Number: 11320082). Each hiPSC was maintained for two passages before pooling into a village using equal numbers of each line. Aliquots of the village were cryopreserved using CryoStor® CS10 (STEMCELL Technologies; Catalog Number: 100–1061) per manufacturer instructions. The village was plated for >90% confluency to start cardiomyocyte differentiations and differentiated over 15 days using the STEMdiff^TM^ Cardiomyocyte Differentiation Kit (STEMCELL Technologies; Catalog Number: 05010) per manufacturer instructions. Cells were collected at Days 0 (hiPSCs at time of village construction), 1, 2, 3, 4, 5, 7, and 15. For hiPSC maintenance interrogations, the cryopreserved village was thawed and plated with mTeSR^TM^ Plus media complete with mTeSR^TM^ Plus Supplement (Life Technologies, Catalog Number: 100–0276) with Rock inhibitor Y-27632 (10 µM final concentration; Stem Cell Technologies; Catalog Number: 72304) on Costar TC-treated six-well plates (Corning; Catalog Number: 3516) coated with Matrigel® hESC-Qualified Matrix (Corning; Catalog Number: 354277) diluted in Dulbecco’s Modified Eagle Medium/F-12 (ThermoFisher Scientific, Catalog Number: 11320082). The villages were maintained for eight passages.

#### scRNA-seq capture

The cardiac differentiated village samples were prepared for the 10x Genomics Single Cell Multiome (ATAC + GEX) kit per manufacturer instructions. Briefly, cells were detached from the plate using TrypleE and washed twice using 0.04% BSA in PBS. Cells were treated with DNAse solution for 5 min on ice and washed using 0.04% BSA in PBS. Cells were counted and 1.5 × 10^6^ cells were used for nuclei extraction. Isolation of nuclei suspensions was performed according to the Demonstrated Protocol: Nuclei Isolation for Single Cell ATAC Sequencing (10x Genomics, CG000365 Rev B) using 0.1× lysis buffer and lysed for 2.5 min to obtain intact nuclei. Single cell ATAC and RNA-seq libraries were prepared using the Chromium single cell multiome ATAC + gene expression platform (10x Genomics). Nuclei were prepared and counted to ensure quality and concentration. Nuclei were then transposed according to the manufacturer’s protocol. Transposed nuclei suspension was loaded onto Next GEM Chip J targeting 20,000 nuclei and then ran on a Chromium Controller instrument to generate GEM emulsion (10x Genomics). Single-cell gene expression libraries, as well as single cell ATAC-seq libraries, were generated according to the manufacturer’s protocol using the Chromium Next GEM Single Cell multiome ATAC+ gene expression kit. Final libraries were quantified using high sensitivity D1000 TapeStation (Agilent). Each library was sequenced separately on a NovaSeq 6000 instrument using an SP 100 cycles reagent kit (Illumina), targeting 25,000 reads/nuclei for ATAC-seq and a minimum of 20,000 reads/nuclei for gene expression

Samples from the hiPSCs villages that were maintained for eight passages—passage one, four and eight—were superloaded onto the 10x Genomics Chromium Controller (10x Genomics) to capture single cells. Briefly, cryopreserved samples were thawed at 37 °C before adding to 4 mL mTeSR^TM^ Plus media complete with mTeSR^TM^ Plus Supplement (Life Technologies, Catalog Number: 100–0276) and centrifuging at 300 × *g* for four minutes to pellet cells. The supernatant was discarded before washing with phosphate-buffered saline (PBS) and incubated in Gibco^TM^ TrypLE^TM^ Express Enzyme (Thermo Fisher Scientific; Catalog Number: 12604-013) for eight minutes. Cold Fluorescence-Activated Cell Sorting (FACS) buffer (PBS with two percent fetal bovine serum [FBS]) was added to the cells before centrifuging at 300 × *g* for four minutes and aspirating the supernatant. The cell pellets were resuspended in 500 µL cold Fluorescence-Activated Cell Sorting (FACS) buffer (PBS with two percent fetal bovine serum [FBS]). Trypan blue was then used to assess the pool viability (>70% viable). Approximately 32,000 cells of each sample were loaded onto a separate well of the Chromium Single Cell Chip B (10x Genomics; Catalog Number: PN-1000073) to capture 20,000 single cells with the Gel Bead Kit V3.0 (10x Genomics; Catalog Number: PN-1000076).

GEM generation, barcoding, cDNA amplification, and library construction were performed according to the 10x Genomics Chromium User Guide (CG000183). Libraries were prepared with the Single Cell 3’ V3.0 Library and Gel Bead Kit (10x Genomics; Catalog Number: PN-1000077 and PN-1000078).

#### Growth rate estimation

The 18 hiPSC lines were each plated in three wells of a 6-well plate on day 0 in mTeSR^TM^ Plus media complete with mTeSR^TM^ Plus Supplement on Costar TC-treated six-well plates coated with Matrigel® hESC-Qualified Matrix diluted in Dulbecco’s Modified Eagle Medium/F-12. Cells were imaged every business day for up to eight days on a Celigo Imaging Cytometer. the media was changed per the manufacturer’s recommendations. The resulting cell confluencies were used to estimate growth rates with *ratrack*^20^.

### scRNA-seq read alignment

The 10x Genomics Cell Ranger Single Cell Software Suite (version 3.1.0) was used to process the 3’ single-cell RNA-seq libraries (chemistry v3). Raw base calls were used to demultiplex the multiplexed pools, which were then mapped to the GRCh38-1.2.0 genome (Ensembl release 84) using STAR (version 2.5.1b) for each pool independently.

### scRNA-seq demultiplexing and doublet detection

The single cell village pools were demultiplexed with *Demuxafy*^21^ to combine droplet calls between different methods. These methods demultiplex the different hiPSC lines in the pools and identify doublets between two lines. The village containing three hiPSC lines used SNP genotype demultiplexing *Popscle Demuxlet v0.1-beta*^13^, *Popscle Freemuxlet v0.1-beta*^22^, *scSplit v1.0.1*^14^, *Souporcell v1.0*^15^, and *Vireo v0.4.2*^12^ with additional doublets classified by *Scrublet*^23^ and *DoubletDetection v 3.0*^24^. Droplets classified as singlets by at least four of the SNP-based demultiplexing or transcription-based doublet detecting softwares, as well as the hashtag demultiplexing and were classified as the same hiPSC line by at least three of the SNP-based demultiplexing softwares, were retained for downstream analysis. All other droplets were excluded.

The cardiomyocyte differentiation of the 18 hiSPC line village was SNP genotype demultiplexed with *Souporcell v1.0*^15^ and additional doublets identified with *scds v1.12.0*^25^ and *DoubletDetection v 3.0*^24^. The village containing 18 hiPSC lines maintained over eight passages was demultiplexed using *Popscle Demuxlet v0.1-beta*^13^, *Popscle Freemuxlet v0.1-beta*^22^, *Souporcell v1.0*^15^, and *Vireo v0.4.2*^12^ with additional doublets classified by *Scrublet v1.0*^23^. The recommended guidelines were followed for each of the softwares as briefly described.

#### Popscle demuxlet

*Popscle pileup* was used to identify the single nucleotide variants (SNVs) in the pool. Then, *Demuxlet* was run with reference genotypes for each hiPSC line in the pool using a genotype error coefficient of 1 and genotype error offset rate of 0.05 and default options for all other parameters.

#### Popscle Freemuxlet

*Popscle pileup* was used to identify the single nucleotide variants (SNVs) in the pool followed by *Freeuxlet* executed with default parameters.

#### scSplit

Low quality and duplicated reads were removed before using freebayes to classify high quality SNVs in the dataset. The resulting bam and vcf were used for *scSplit* using default options and the -n 3 option to provide the number of hiPSC lines in the pool.

#### Souporcell

*Souporcell* was run using the *souporcell_pipeline.py* script with known variant locations from the reference imputed SNP genotypes that overlapped gene exons using the*–common_variants* parameter and all other default parameter options.

#### Vireo

Model 1 of cellSNP v0.3.2 was used to identify allele frequencies at the locations of the common variants (MAF = 0.1) in the genotyped reference genotype file for the three hiPSC lines. The resulting pileup was filtered for SNP locations covered by at least 20 UMIs and had at least 10% minor allele frequency across all droplets. Vireo version 0.4.2 was then used to demultiplex the droplets in the pool using reference SNP genotypes and indicating the number of individuals in the pools.

#### Scrublet

*Scrublet* was used to identify transcription-based doublets that included two cells from different cell types. *Scrublet* was implemented in *python v3.6.3* per developer recommendations with at least three counts per droplet, three cells expressing a given gene, 30 PCs and a doublet rate based on the following equation:

1 $$R=\frac{{N}^{2} \,*\, 0.008}{1000}$$

where *N* is the number of droplets captured and *R* is the expected doublet rate. *Scrublet* was assessed at four different minimum numbers of variable gene percentiles: 80, 85, 90, and 95. Then, the best variable gene percentile was selected based on the distribution of the simulated doublet scores and the location of the doublet threshold selection. If the chosen threshold does not fall between a bimodal distribution, those pools were rerun with a manual threshold set.

#### Scds

*Scds*^25^ is a transcription-based doublet detecting method. *Scds* was implemented with the *cxds* function and *bcds* functions with default options followed by the *cxds_bcds_hybrid* with *estNdbl* set to TRUE so that doublets were estimated based on the values from the *cxds* and *bcds* functions.

#### DoubletDetection

*DoubletDetection*^24^ is a transcription-based method for identifying doublets. Droplets without any UMIs were removed before analysis with *DoubletDetection*. Then the *doubletdetection.BoostClassifier* function was run with 50 iterations with *use_phenograph* set to False and *standard_scaling* set to True. The predicted number of doublets per iteration was visualized across all iterations. Any pool that did not converge after 50 iterations were rerun with increasing numbers of iterations until they reached convergence.

#### Hashtag demultiplexing

Hashtag demultiplexing^26^ was used to identify cells from each location and doublets that included cells from two locations.

### Quality control

#### Three-line village

144,988 droplets were captured for analysis. Droplets were considered outliers and excluded from further analysis if they were more than four median average deviations (MAD) from the mitochondrial percentage median or contained <1750 total genes. This resulted in 88,927 high-quality single cells being used for downstream analysis. Cyclone from the scran package v1.4.5^27^ was used to detect the cell cycle state of each cell. The quality control metrics for these high-quality single cells for the fresh samples (Fig. 2a) are presented in Supplementary Fig. 4, and the cryopreserved experiment samples (Fig. 3a) are presented in Supplementary Fig. 5.

Data were normalized with a regularized negative binomial regression, and variance stabilized with Pearson residuals using *SCTransform* as previously described^28^. Expression data were also corrected by cell cycle status, mitochondrial percentage, and ribosomal percentage.

#### Eighteen-line village

57,451 droplets were captured, and 29,055 cells remained after removing the droplets that were classified as a doublet by *Demuxafy* using the ‘AtLeastHalfSinglet’ integration method (28,081 droplets removed) and those with >25% mitochondrial content (315 additional droplets removed).

### Correlation of transcriptional profiles

The mean expression of each gene for each line was compared between samples with a two-sided Spearman Rank correlation, and the distributions of the correlations between samples of different covariates were plotted.

### hiSPC line proportional changes between samples

The proportional changes of lines between samples were estimated using *scCODA*^29^ v0.1.8. An FDR of 0.05 was used for all *scCODA* analyses. *scCODA* was sequentially run once using each cell line as the reference for each analysis and the majority vote was used to identify cell lines whose proportion was credibly changed more than half the time. The specific metrics are provided in Supplementary Data 8).

### Correlation of growth rates with hiPSC line proportions − 18-line hiPSC Village

The growth rates of the hiPSC lines in the 18-line village that were estimated with ratrack^20^ were correlated with proportions of hiPSC line estimated using *Demuxafy* at each time single cells were captured. Two-sided Spearman rank correlation was used to measure the strength of the correlation and statistical significance.

### Covariate contribution to gene variance

The proportion of the variance explained by the hiPSC line, the replicate and the village status for each gene was determined by fitting a linear mixed model for normalized and regularized expression of each gene. Briefly, normalized UMI counts were fit as the dependent variable of a linear mixed model with the independent variables as random effects. The intra-class correlation (ICC) was used to estimate the variance explained by each variable where *var* is the estimated variable, *i*:*n* is all the variables, including residual and *σ*^2^ represents the estimated variance.

2 $${{{{{\rm{ICC}}}}}}=\frac{{{\sigma }}_{{{{var}}}}^{2}}{{\sum }_{i}^{n}{{\sigma }}_{i}^{2}} \,*\, 100$$

The variables included for the fresh samples across the three sites were: line, village, site, and replicate. For the cryopreserved samples, the variables were line, village, cryopreservation and replicate. For the village containing 18 lines, the covariates modeled were line and passage number. The significance of a given variable was tested using an ANOVA between the model with and without that covariate. Significant covariates were then tested for significant interaction as well.

### Pluripotent gene differential expression

All genes expressed in at least 10% of cells in a given line at a given site were tested for differential expression with a logistic regression between the uni-culture and village samples. For comparison across the samples, all groups were down-sampled to the smallest number of cells at one condition (*n* = 572). Differential expression was detected using logistic regression implemented in Seurat. Replicates were fit as a covariate in the model, and significance was corrected for all tests across all groups with the Bonferroni correction method.

### hiPSC eQTL replication

eQTLs previously identified by DeBoever et al. with bulk RNA-seq in hiPSCs were filtered for SNPs with at least two alleles across the three lines used in the village and genes with a significant portion of their variance explained lines. Those SNPs were then filtered for SNPs with non-identical alleles in the three lines and tested for effects with a linear model fitting the gene expression. The average expression for each hiPSC line at each site were fit for the uni-culture and village samples separately with the covariates previously identified to contribute to the gene expression variance (see “Covariate contribution to gene variance” section). The effects (β) were tested for significant agreement with DeBoever et al. eQTLs with a χ^2^ test. The correlation and significance between uni-culture and village effect sizes was calculated using a two-sided Spearman rank correlation.

### RNA velocity pseudotime

Pseudotime was estimated using RNA velocity implemented with the *scvelo*^30^ package (v0.2.3) to estimate the latent time of all single cells. First, sequence reads overlapping spliced and unspliced read count matrices were prepared using *velocyto*^31^ (v0.17.17). Cells that had <1000 unspliced counts and genes that were expressed in <20 cells and had <10 unspliced counts were filtered and removed. The batch effects were normalized using *pycombat*^32^ (*Combat v0.3.0*) using a proposed approach^33^. Briefly, the spliced ($S$) and unspliced ($U$) counts were combined to create the total count matrix ($M;$Eq. 3). An additional matrix ($R$) was also constructed to aid in deriving the corrected spliced (${S}_{b}$) and unspliced (${U}_{b}$) matrices following batch correction of $M$ (Eq. 4). Then M was corrected for site, hiPSC line and village status batch effects using *pycombat*. Following batch correction, batch-corrected splicted (${S}_{b}$) and unspliced (${U}_{b}$) matrices were derived using the $R$ matrix (Eqs. 5 and 6).

3 $$\,M=S+U$$

4 $$\,R\,=\frac{S}{S+U}$$

5 $${{S}_{b}=M}_{b} \,*\, R$$

6 $${U}_{b}={M}_{b} \,*\, \left(1-R\right)$$

Those matrices were then used to calculate the dynamical RNA velocity latent time with the *scvelo* package.

### Gene expression variance explained by pseudotime

The gene expression variance explained by pseudotime was modeled with a linear mixed model described in “Covariate contribution to gene variance” with pseudotime as a continuous covariate. The interaction of pseudotime and line effect was tested if both the line and pseudotime were significant contributors to gene expression variance.

### Integrating conditions for visualization

Cells from each cell line in each village status and at each site were integrated for visualization (Fig. 4b and Supplementary Fig. 6b) using the reciprocal principal component analysis (RPCA) method implemented with the *Seurat* package (v4.0.0-4.0.5)^34^. Thirty PCs, cell cycle, and mitochondrial percentage were fit as covariates.

### Statistics and reproducibility

#### 3-line hiPSC village

Three hiPSC lines that were previously established, well-characterized and used in previous studies were selected for use in the three-line hiPSC village experiments described. Three hiPSC lines, three sites and three replicates at each site were selected as the minimum required number to effectively identify variation between the different covariates. Four days of culture in the hiPSC villages were selected since this is typical for seeding stem cells in preparation for differentiation experiments. One week was used for the cryopreservation experiments since at least one week is required for stem cells to recover from thawing before beginning experiments. Single cell capture pools were generated with one sample from each site to prevent possible technical variation during capture, library preparation and sequencing that could confound site effects. Only droplets classified to contain cells from the cellranger pipeline were used in subsequent analyses. The resulting data were demultiplexed with genetic data (for each hiPSC line) and antibody hashtags (for each site). Droplets identified as doublets (containing two or more cells) or those could not be assigned to either a site or a hiPSC line were removed from the analysis. Data were then filtered for cells that were less than four median average deviations (MAD) from the mitochondrial percentage median or contained more than 1750 total genes (Supplementary Figs. 10, 11). Statistical analyses were conducted in R and python using established tools or methods with scripts for all analyses provided on Github (https://github.com/powellgenomicslab/iPSC_Village_Publication) and Zenodo^35^. Data have been made available to support the reproduction of these results on Gene Expression Omnibus (GSE225282) and Zenodo^35^. Growth rate experiments were blinded to the experimenter. Experiments were not randomized.

### 18-line hiPSC village

#### Cardiomyocyte 18-line village

No statistical method was used to predetermine the sample size. The hiPSC villages were captured on Days 0, 1, 2, 3, 4, 5, 7, and 15 to capture the transcriptional changes that occur early during differentiation. Droplets that contained cells based on cellranger estimation were maintained for analysis. Further, droplets that were annotated as doublets or could not be assigned to a hiPSC line with genetics by demultiplexing and/or doublet detecting methods were removed from downstream analyses. Growth rate experiments were blinded to the experimenter. Experiments were not randomized.

#### Multi-passage 18-line village

No statistical method was used to predetermine sample size. The hiPSC villages were cryopreserved after one, four or eight passages and then thawed and captured at the same time to prevent possible capture, library preparation or sequencing confounding effects. Droplets that were not identified to contain cells by cellranger were removed from downstream analyses. Further, droplets that could not be assigned to a single hiPSC line with genetic demultiplexing were also removed. The growth rate experiments were blinded to the experimenter. The experiments were not randomized.

All analyses were executed in R version 4.2.1 or python versions > 3.6.8

### Reporting summary

Further information on research design is available in the Nature Portfolio Reporting Summary linked to this article.

## Supplementary information

Supplementary information

Description of Additional Supplementary Files

Supplementary Data 1

Supplementary Data 2

Supplementary Data 3

Supplementary Data 4

Supplementary Data 5

Supplementary Data 6

Supplementary Data 7

Supplementary Data 8

Reporting Summary

## Data Availability

The raw and semi-processed single-cell and genetic data are available on Gene Expression Omnibus under accession code “GSE225282”. Completely processed Seurat objects and other data are available on Zenodo^35^. All other relevant data supporting the key findings of this study are available within the article and its Supplementary Information files or from the corresponding author upon reasonable request. Source data are provided with this paper.

## Source data

Source data

## Code availability

Analysis code is available on Github (https://github.com/powellgenomicslab/iPSC_Village_Publication) and Zenodo^35^.
