# Supplementary material for: A village in a dish model system for population-scale hiPSC studies
Source: Nat Commun. 2023 Jun 9;14:3240. doi: 10.1038/s41467-023-38704-1 (PMC10256711; doi:10.1038/s41467-023-38704-1)
Supplement: Supplementary file 1 — Supplementary information [file 41467_2023_38704_MOESM1_ESM.pdf]

Supplementary Figures for:

Village In a Dish: A Model System for Population-scale hiPSC Studies

Drew R. Neavin[1], Angela M. Steinmann[1]*, Nona Farbehi[1,2]*, Han Sheng Chiu[3]*, Maciej S. Daniszewski[4]*, Himanshi Arora[1], Yasmin Bermudez[1], Cátia Moutinho[1], Chia-Ling Chan[1], Monique Bax[5-6], Mubarika Tyebally[1], Vikkitharan Gnanasambandapillai[1], Chuan E. Lam[1], Uyen Nguyen[1], Damián Hernández[4], Grace E. Lidgerwood[4], Bob Graham[5-7], Alex W. Hewitt[8,9,13], Alice Pébay[4,10,13], Nathan J. Palpant[3,13], and Joseph E. Powell[1,11,12,13]

1. Garvan-Weizmann Centre for Cellular Genomics, Garvan Institute of Medical Research, Darlinghurst, 2010, Sydney
2. Graduate School of Biomedical Engineering, University of New South Wales, Kensington, 2033, Sydney
3. Institute for Molecular Bioscience, University of Queensland, Brisbane
4. Department of Anatomy and Physiology, the University of Melbourne, Australia
5. Victor Chang Cardiac Research Institute, Darlinghurst, NSW, Australia
6. UNSW Medicine & Health, UNSW Sydney, Kensington, NSW, Australia
7. St Vincent's Hospital, Darlinghurst, 2010, NSW, Australia
8. Centre for Eye Research Australia, Royal Victorian Eye and Ear Hospital, University of Melbourne, Melbourne, Australia
9. School of Medicine, Menzies Institute for Medical Research, University of Tasmania, Hobart, Australia
10. Department of Surgery, Royal Melbourne Hospital, Anatomy and Neuroscience, the University of Melbourne, Australia
11. UNSW Cellular Genomics Futures Institute, School of Medical Sciences, University of New South Wales, 2052, Sydney
12. Correspondence: j.powell@garvan.org.au
13. These authors jointly supervised this work

* these authors contributed equally

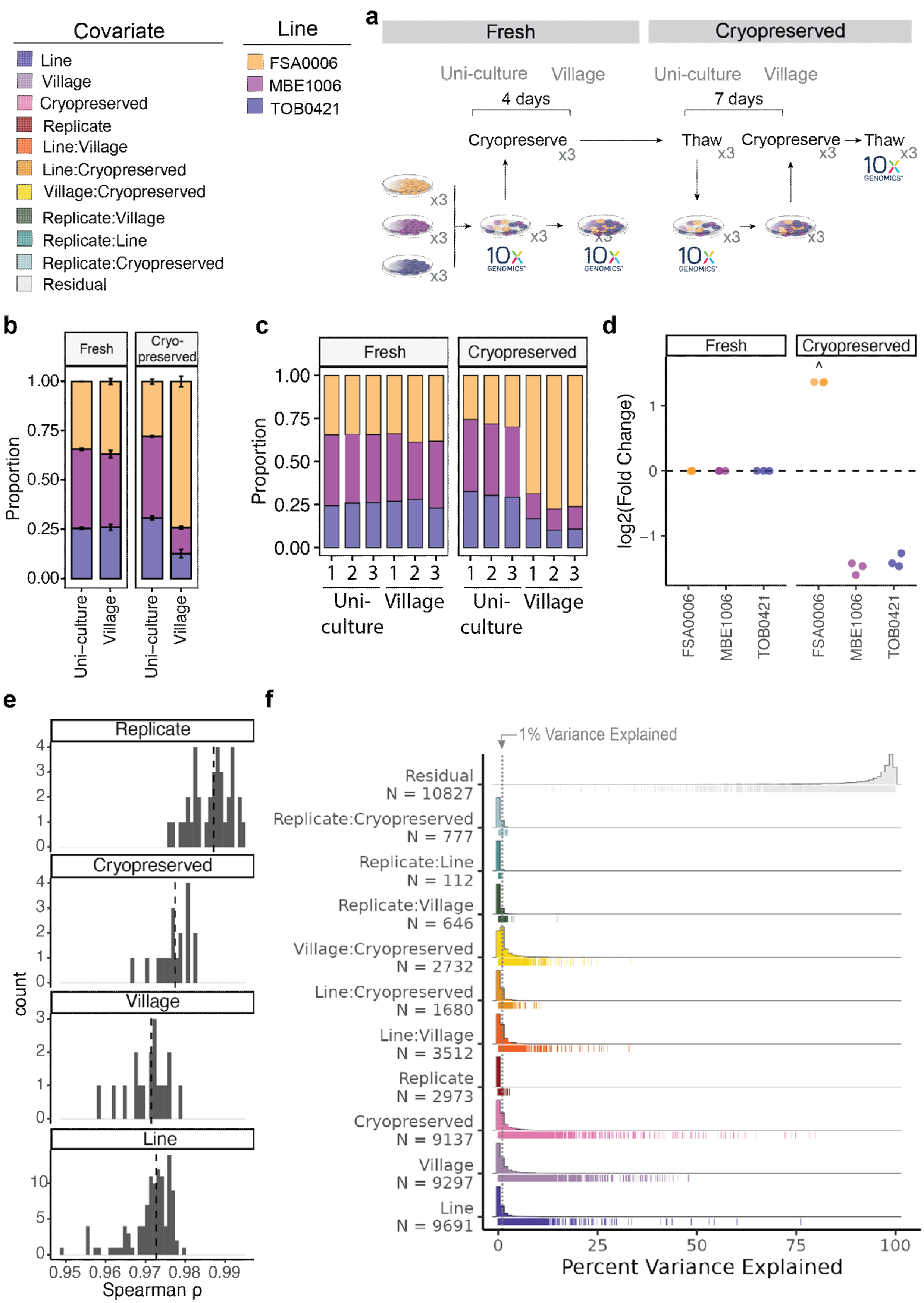

Covariate
Line
Village
Cryopreserved
Replicate
Line:Village
Line:Cryopreserved
Village:Cryopreserved
Replicate:Village
Replicate:Line
Replicate:Cryopreserved
Residual
Line
FSA0006
MBE1006
TOB0421
a
Fresh
Cryopreserved
Uni-culture
Village
4 days
7 days
Cryopreserve
Thaw
x3
10x GENOMICS
b
Fresh
Cryo-preserved
Proportion
Uni-culture
Village
c
Proportion
1 2 3 1 2 3
Uni-culture
d
log2(Fold Change)
e
Replicate
count
Spearman ρ
f
1% Variance Explained
Residual N = 10827
Replicate:Cryopreserved N = 777
Replicate:Line N = 112
Replicate:Village N = 646
Village:Cryopreserved N = 2732
Line:Cryopreserved N = 1680
Line:Village N = 3512
Replicate N = 2973
Cryopreserved N = 9137
Village N = 9297
Line N = 9691
Percent Variance Explained

Supplementary Figure 1: Impact of village culturing systems on cell numbers and transcriptional profiles. a) Experimental design to test impact of village culturing systems on hiPSC transcriptional profiles following cryopreservation. **b)** Proportion of each hiPSC line in uni-culture or village at each site. Error bars show the standard error of the triplicates. N = 3 replicates at each site for uni-culture and village. **c)** Proportion of each hiPSC line in uni-culture or village at each site split by replicate. **d)** scCODA assessment of differential hiPSC line proportions for each condition demonstrates that the proportions of all FSA0006 changed during village culturing in the cryopreserved samples but not the fresh samples. N = 3 replicates at each site for uni-culture and village. **e**) Correlations between samples for each covariate - Replicate, Village, Line and Site. Dashed lines represent the median of the correlations between samples for the given covariate. f) Variance of gene expression explained by each covariate. The lines below the histogram represent a gene for each covariate and the dashed line indicates 1% of variance explained. hiPSC: human pluripotent stem cell; ^: scCODA-detected proportional change.

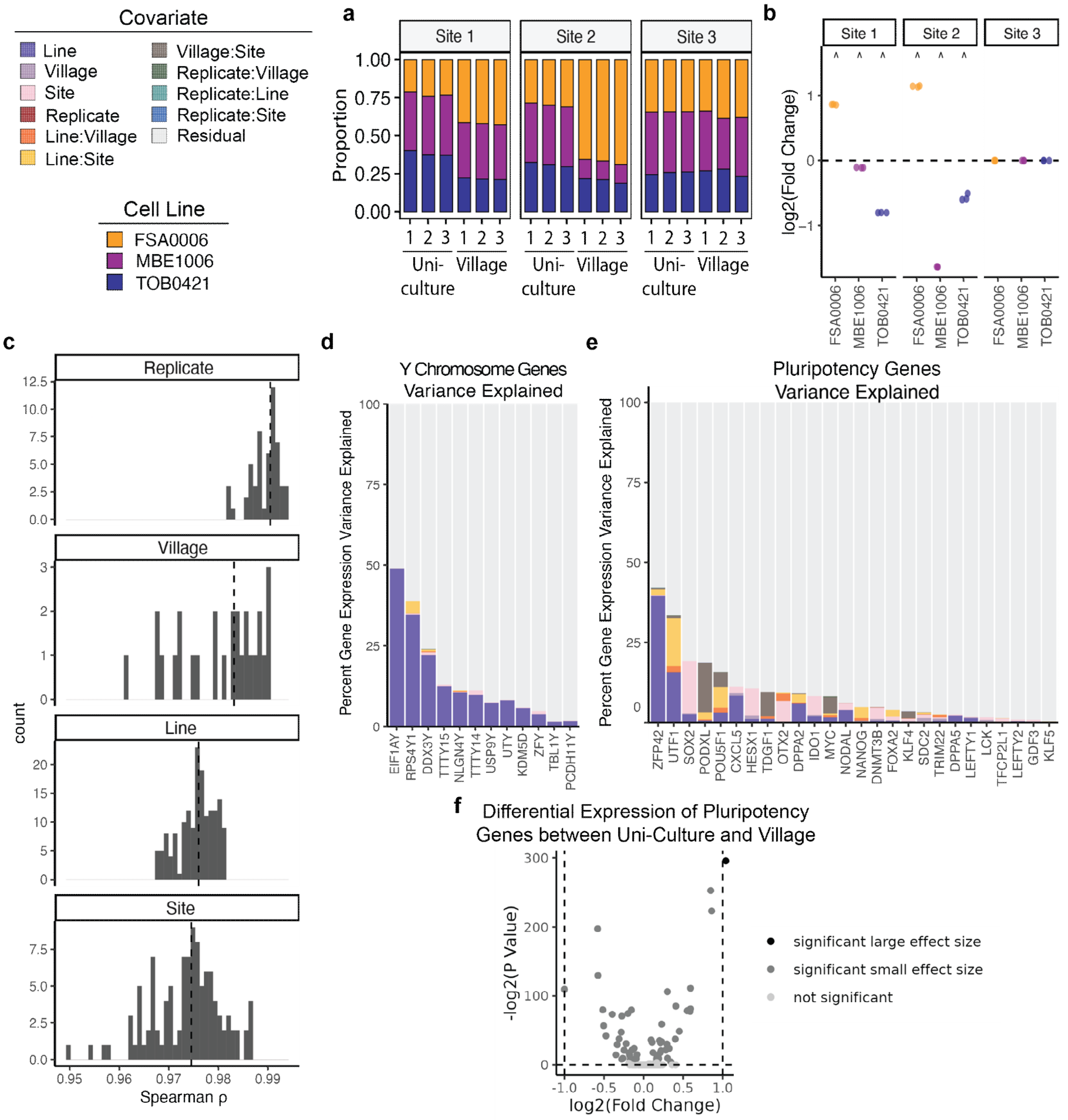

Supplementary Figure 2: Impact of village culturing systems on cell numbers and transcriptional profiles. a) Proportion of each hiPSC line in each replicate in uni-culture or village at each site. **b)** scCODA assessment of differential hiPSC line proportions for each condition demonstrates that the proportions of all hiPSC lines changed at Sites 1 and 2 but not 3. N = 3 replicates at each site for uni-culture and village. **c**) Correlations between samples for each covariate - Replicate, Village, Line and Site. Dashed lines represent the median of the correlations between samples for the given covariate. **d**) Proportion of Y chromosome genes explained by the covariates measured in this dataset. **e**) Proportion of Pluripotency genes explained by the covariates measured in this dataset. **f**) Volcano plot of the differential expression of pluripotency genes between uni-culture and village samples estimated using a logistic regression and

corrected for multiple comparisons. Most significant differentially expressed genes had small effect sizes. hiPSC: human pluripotent stem cell; ^: scCODA-detected proportional change;

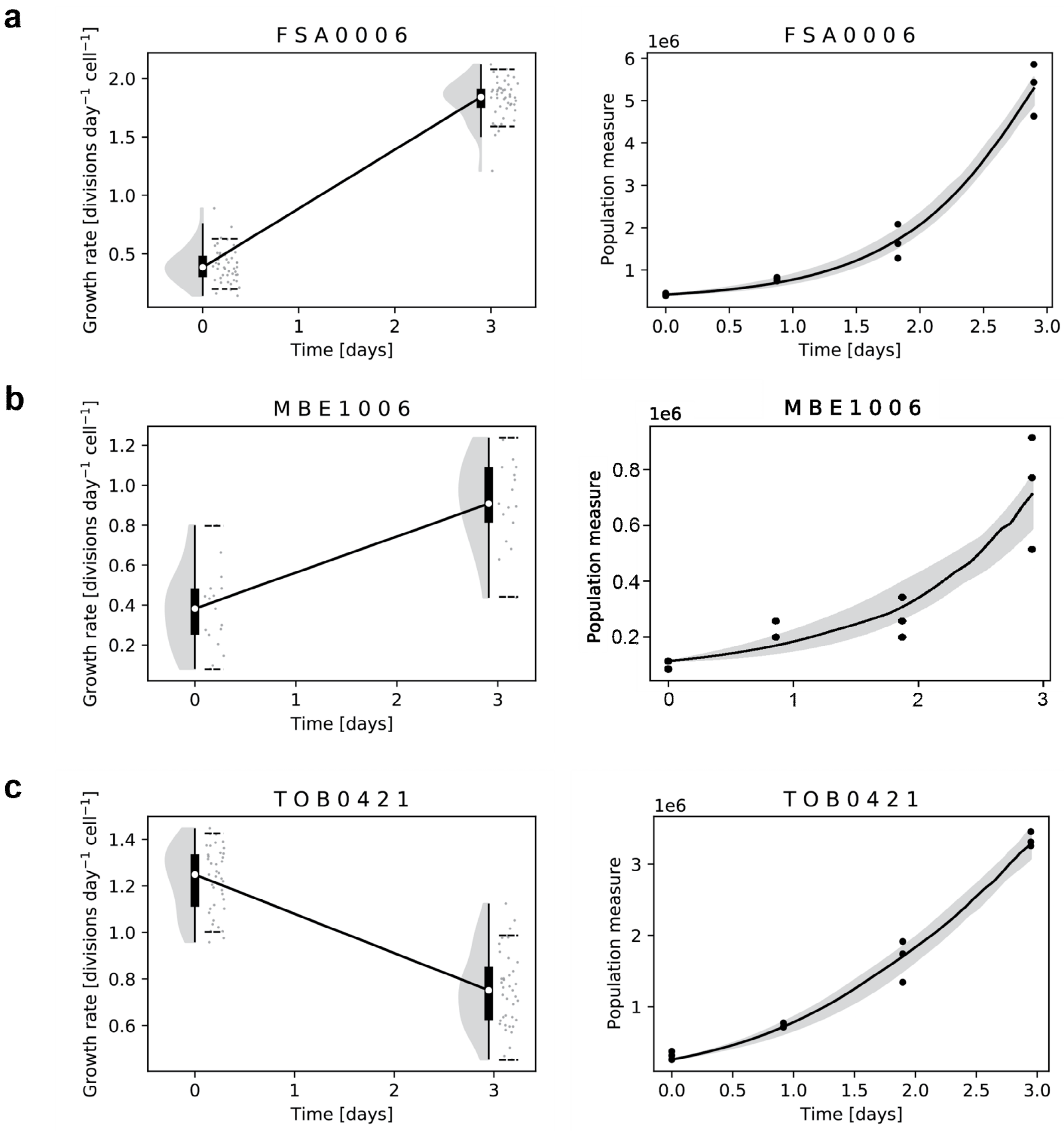

Supplementary Figure 3: hiPSC line growth rates. The growth rate of FSA0006 (**a**) is larger than the growth rates for MBE1006 (**b**) or TOB0421 (**c**). The grey distributions (left) indicate the density of the fitted growth rates and the grey bands around the fitted growth line (right) indicate the standard error of the estimated growth rate at that time. The box plots (left) show the median as the center line, upper and lower quartiles and the box limits, the range as the whiskers with the

1.5x interquartile ranges as the dotted lines to the right of the box plots. N = 3 replicates on each day.

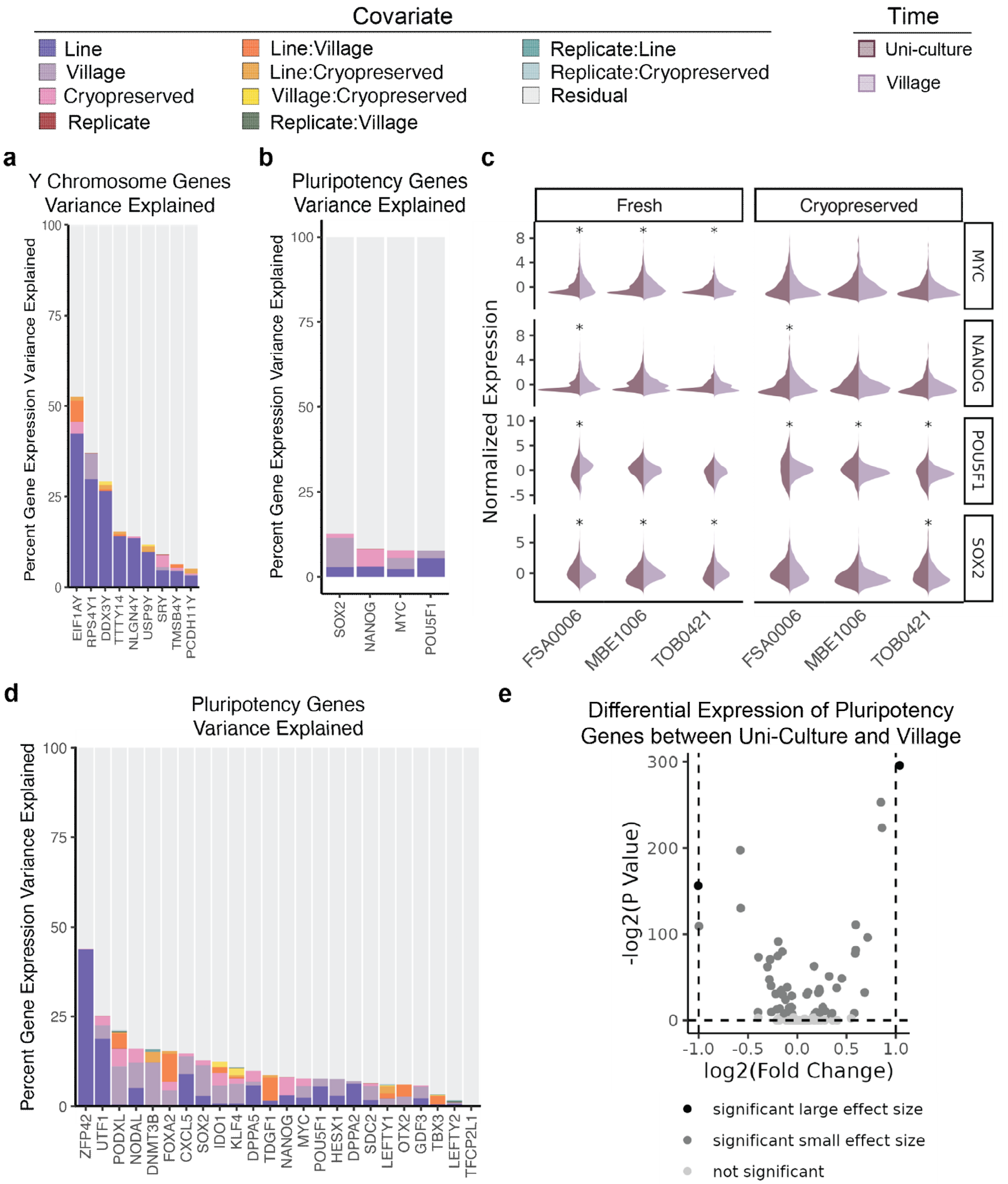

Supplementary Figure 4: Impact of village culturing system on cryopreserved samples. a) Proportion of Y chromosome genes explained by the covariates measured in this dataset. **b**) Proportion of four important pluripotency genes explained by the covariates measured in this dataset. **c**) Expression of the four important hiPSC markers. Asterisks indicate significant differential expression between the uni-cultiure and village samples estimated using a logistic regression and corrected for multiple comparisons. **d**) Variance of the gene expression of a larger number of hiPSC markers by the covariates measured in to compare fresh and cryopreserved uni-culture and village samples. **e**) Differential expression of the pluripotency markers. Although significant, most have relatively small fold change differences between uni-culture and village samples. Differential expression was estimated with a logistic regression and p-values were corrected for multiple comparisons. hiPSC: human pluripotent stem cell; ^: scCODA-detected proportional change; *: adjusted P-value < 0.05.

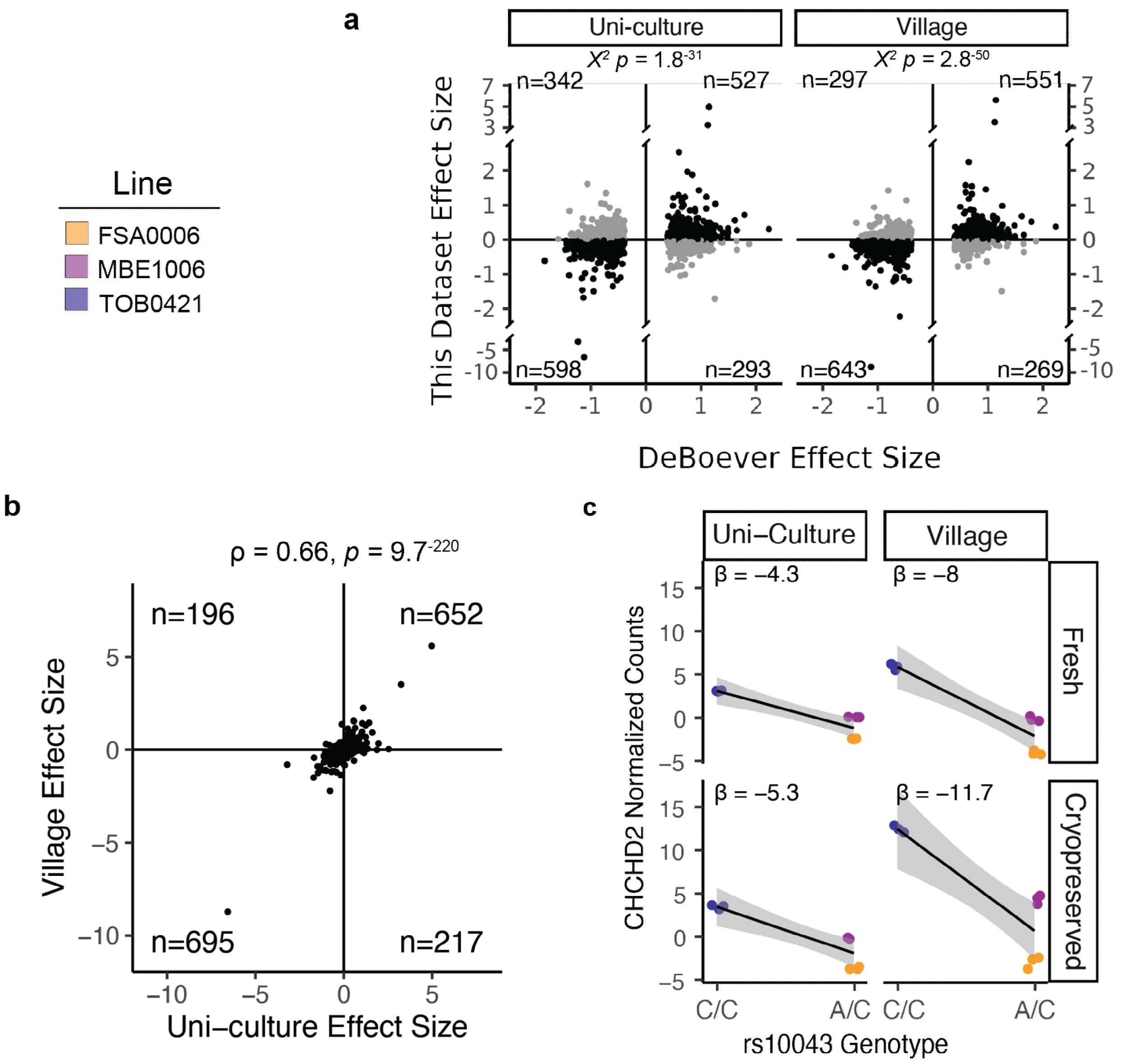

Supplementary Figure 5: eQTL detection consistent in Uni-culture and Village samples. a) Reproduction of eQTLs previously described by DeBoever *et al*. Significance detected with two-sided Chi-squared test. **b**) Two-sided Spearman Rank Correlation between the effect sizes of previously-reported eQTLs in the Uni-culture (x-axis) and Village (y-axis) samples. **c)** The previously-reported eQTL for *CHCHD2* demonstrates a strong and consistent effect across different Sites and the Village status. The grey band around the line indicates the standard error.

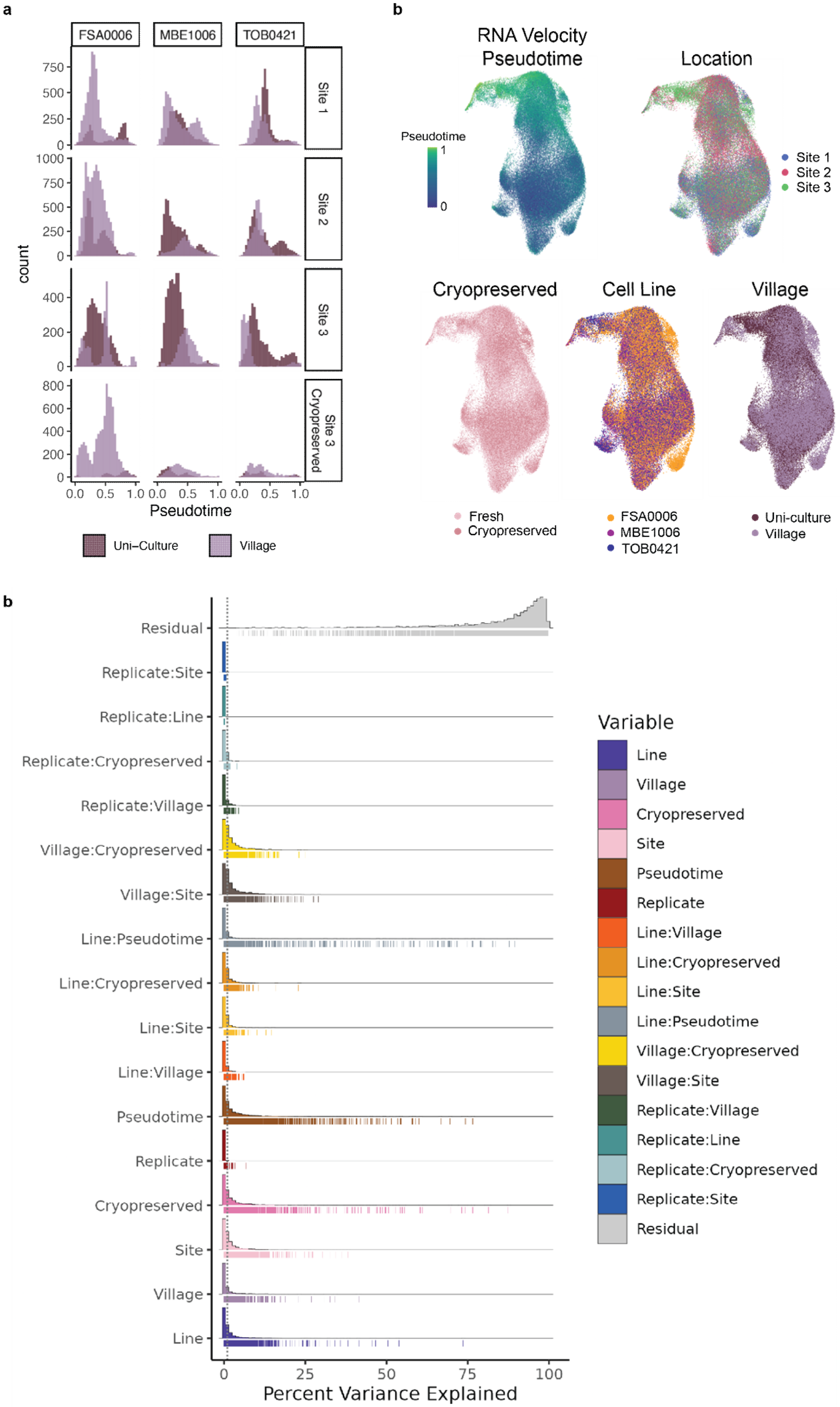

a
FSA0006
MBE1006
TOB0421
Site 1
Site 2
Site 3
Site 3 Cryopreserved
count
Pseudotime
Uni–Culture
Village
b
RNA Velocity Pseudotime
Location
Pseudotime
Site 1
Site 2
Site 3
Cryopreserved
Cell Line
Village
Fresh
Cryopreserved
FSA0006
MBE1006
TOB0421
Uni-culture
Village
b
Residual
Replicate:Site
Replicate:Line
Replicate:Cryopreserved
Replicate:Village
Village:Cryopreserved
Village:Site
Line:Pseudotime
Line:Cryopreserved
Line:Site
Line:Village
Pseudotime
Replicate
Cryopreserved
Site
Village
Line
Percent Variance Explained
Variable
Line
Village
Cryopreserved
Site
Pseudotime
Replicate
Line:Village
Line:Cryopreserved
Line:Site
Line:Pseudotime
Village:Cryopreserved
Village:Site
Replicate:Village
Replicate:Line
Replicate:Cryopreserved
Replicate:Site
Residual

Supplementary Figure 6: Dynamic variance explained across pseudotime. a) Distributions of the pseudotime of the cells from each hiPSC line at each site. **b**) UMAP plots colored by the pseudotime, site, cryopreservation status, hiPSC line and village status. **c**) The distributions of variance explained by each of the covariates. Each line in the rug plot below the histrograms indicates a different gene for the given covariate. hiPSC: human induced pluripotent stem cell.

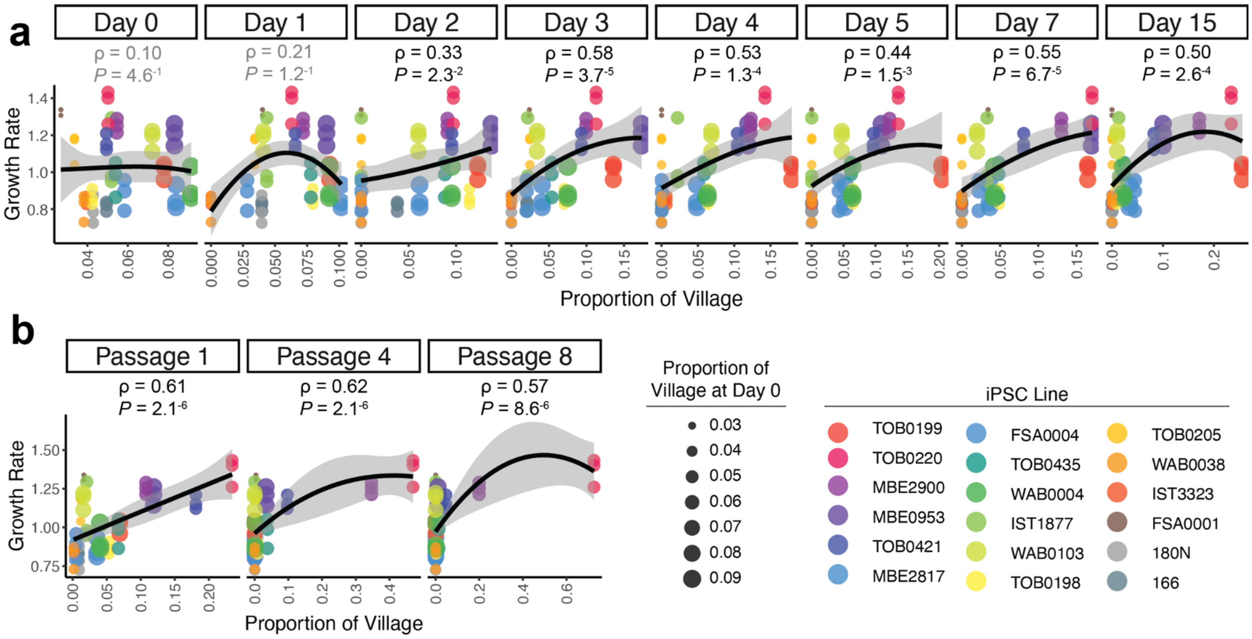

Supplementary Figure 7: iPSC growth rates reflect proportion of cells in villages. a) Correlation of the growth rate of each iPSC line to the proportion of cells measured in villages during cardiomyocyte differentiation. As expected, the growth rates are not correlated at Day 0 but demonstrate stronger correlations with each day until Day 3 when the correlations maintain a similar strength for the remainder of the differentiation. **b**) The growth rate of each iPSC line is strongly correlated to the proportion of cells measured in villages across multiple passaged. Significant correlations are in black and unsignificant in grey. Correlations measured with two-sided Spearman Rank Correlation. P-values are Benjamini-Hochberg corrected.

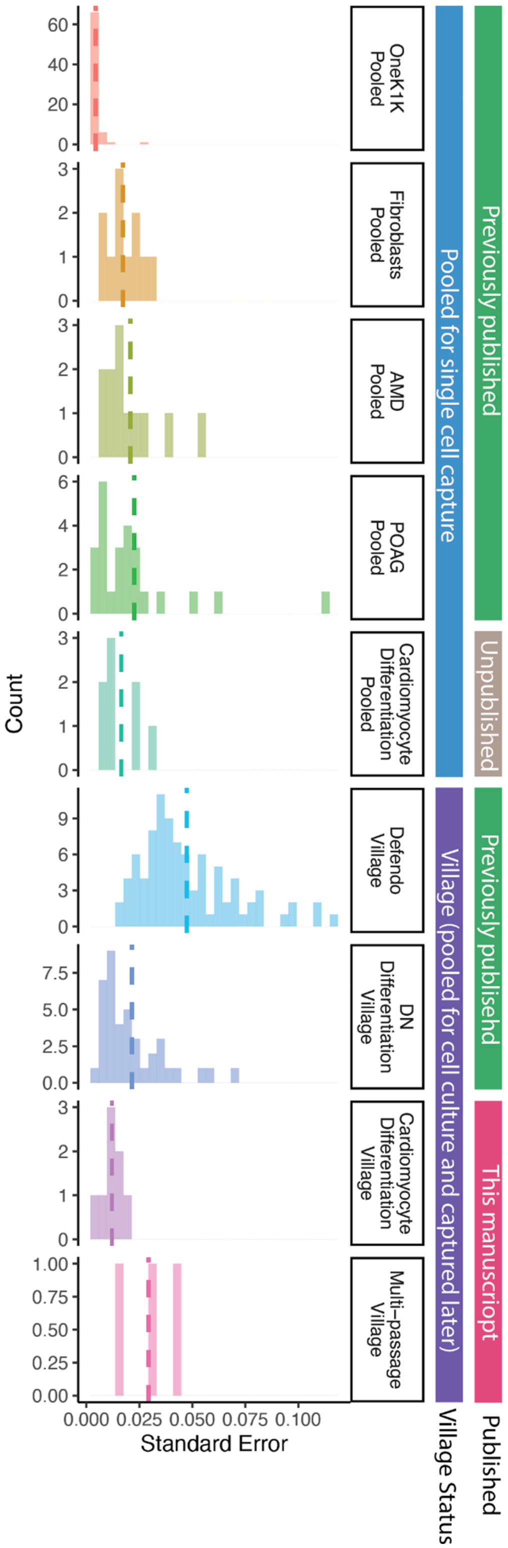

Supplementary Figure 8: Distribution of variances of cell numbers per sample for pooled single cell captures. Distribution of the standard error of numbers of cells from each individual for pooled single cell captures for multiple different cell types and experimental designs. The variance of the numbers of cells per iPSC line in the Cardiomyocyte Differentiation Village and

Multi-passage Village datasets from this paper are within the expected distributions of previous datasets. The datasets used for comparison included ‘Pooled’ designs where cells from unrelated donors were pooled at the time of single cell capture and ‘Village’ designs where cells from unrelated donors were pooled and cultured together to be captured with single cell methods at a later date (blue and purple bars, respectively). These variance distributions represent diverse cells types including peripheral blood mononuclear cells (OneK1K Pooled), fibroblasts (Fibroblasts Pooled), iPSC-derived retinal epithelium from donors with age-related macular degeneration (AMD Pooled), iPSC-derived retinal organoids from patients with primary open-angle glaucoma (POAG Pooled), iPSC-derived cardiomyocytes (Cardiomyocyte Differentiation Pooled and Cardiomyocyte Differentiation Village), iPSC-derived definitive endoderm differentiation (Defendo Village), iPSC-derived dopaminergic neuron differentiation (DN Village) and iPSCs (Multi-passage Village). The dashed line represents the average standard error for each dataset. iPSC: induced pluripotent stem cell.

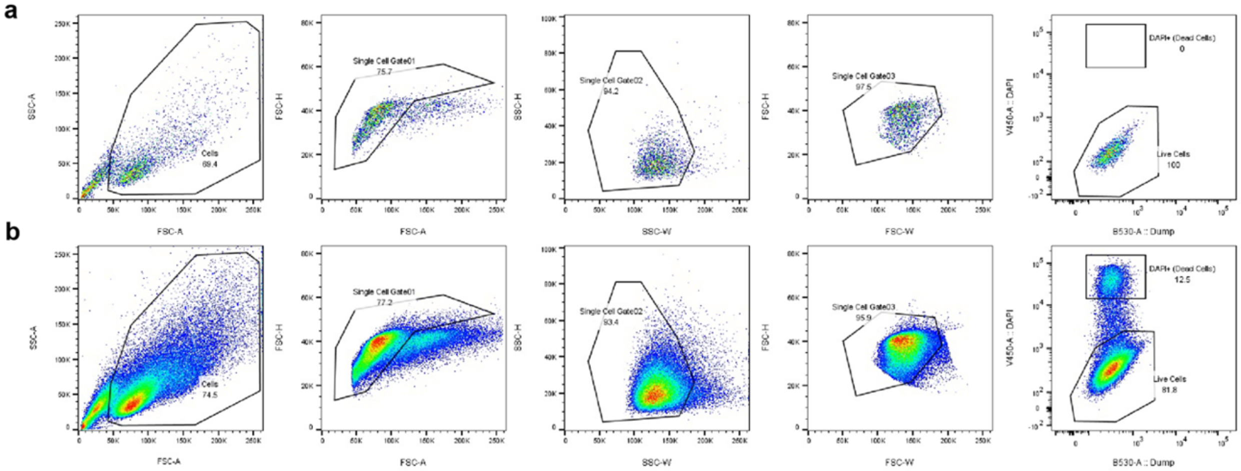

Supplementary Figure 9: Fluorescence-Activated Cell Sorting Gating. a) Cell suspension without DAPI to gate for cells, then single cells. The DAPI gating demonstrate no cells with DAPI positive when the cells were unstained with DAPI. **b)** Cell suspension stained with DAPI gated for cells with side and forward scatter area followed by identification of single cells with a combination of forward scatter height, weight and area and side scatter height, weight and area. Live cells were gated from the single cell population with V540A. DAPI: 4′,6-diamidino-2-phenylindole (DAPI).

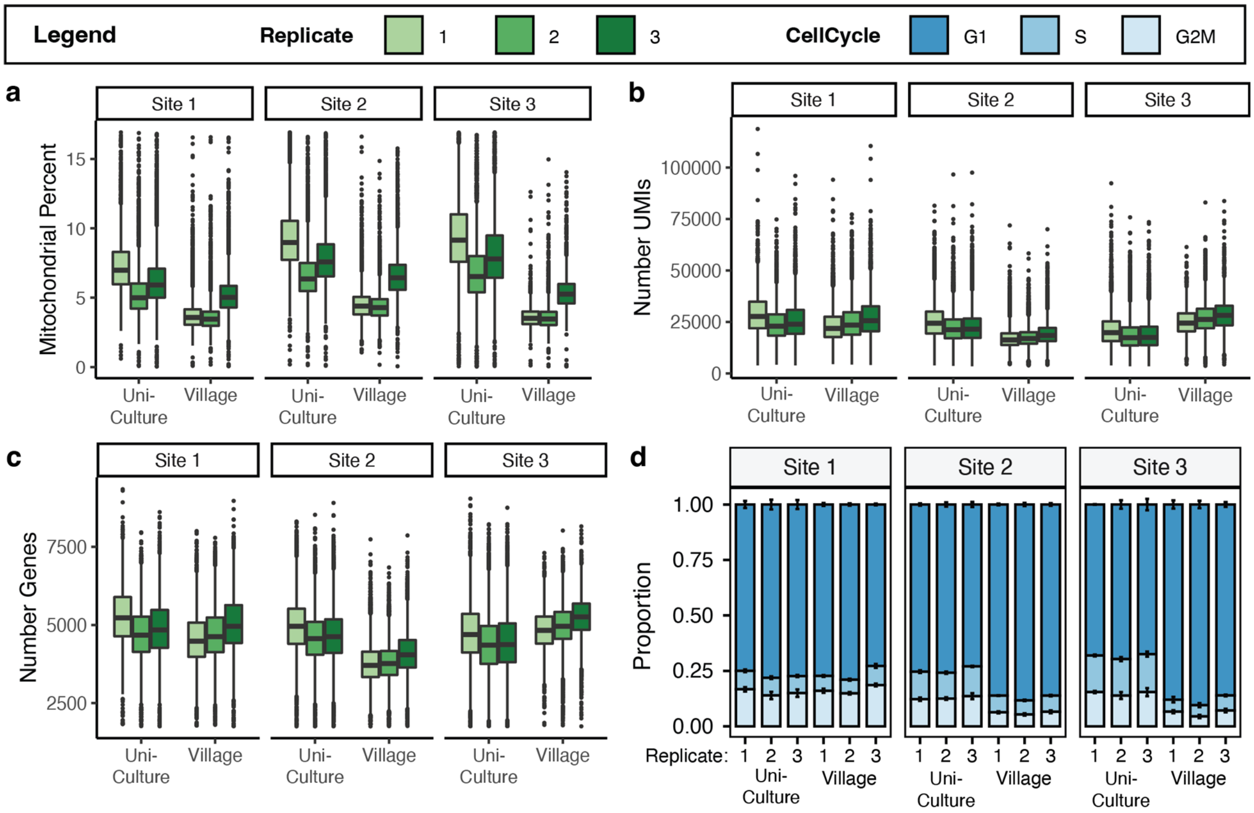

Supplementary Figure 10: Quality Control Metrics at Three Sites. a) The mitochondrial percent was different between baseline and village cultured cells but relatively consistent between the sites. **b-c)** The number of UMIs and number of genes detected is relatively consistent between Sites and village status. **d)** The proportions of cells in each cell cycle group was relatively consistent between baseline and village culturing conditions for Site 1 but there were more cells in G1 and less in S and G2M phases for Sites 2 and 3. The box plots (**a**-**c**) show the median as the center line, upper and lower quartiles ads the box limits, 1.5x interquartile ranges as the whiskers and outliers as the points. The error bars on the bar plot (**d**) show the standard error. N = 75,571 cells examined across the three sites and uni-culture or village.

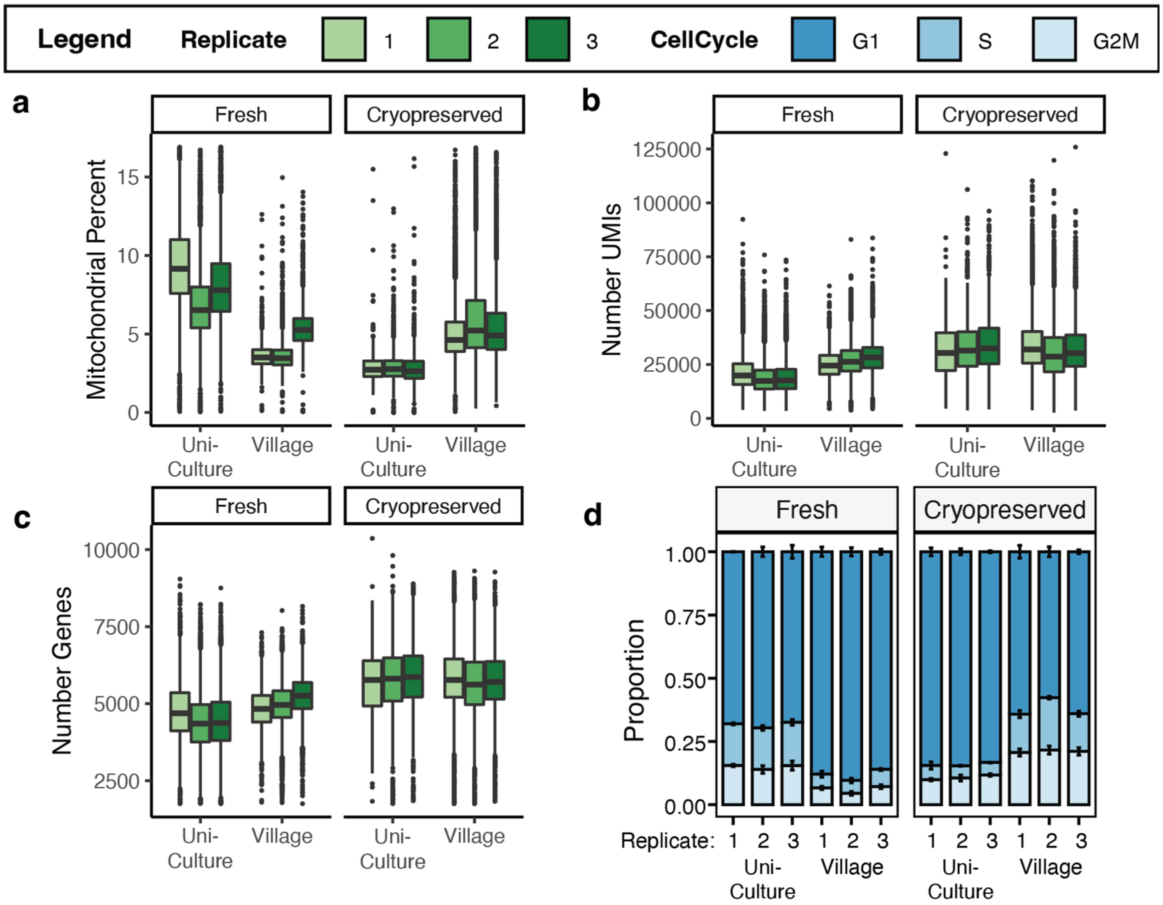

Supplementary Figure 11: Quality Control Metrics for Fresh and Cryopreserved Samples. a) The mitochondrial percent were not consistent for cells collected at baseline or village statuses or between fresh and cryopreserved samples. **b-c)** The number of UMIs and genes were relatively consistent between baseline and village samples but were higher in the cryopreserved samples. **d)** The proportions of cell cycle groups were consistent between replicates in each condition but inconsistent between conditions. The box plots (**a**-**c**) show the median as the center line, upper and lower quartiles and the box limits, 1.5x interquartile ranges as the whiskers and outliers as the points. The error bars on the bar plot (**d**) show the standard error of all cells for that replicate and condition. N = 31,264 cells examined across the three sites and uni-culture or village.
