# Supplementary material for: A village in a dish model system for population-scale hiPSC studies
Source: Nat Commun. 2023 Jun 9;14:3240. doi: 10.1038/s41467-023-38704-1 (PMC10256711; doi:10.1038/s41467-023-38704-1)
Supplement: Supplementary file 2 — Description of Additional Supplementary Files [file 41467_2023_38704_MOESM2_ESM.pdf]

## Description of Additional Supplementary Files

**Supplementary Data 1**
Variance of gene expression explained by each of the covariates for uni-culture and village samples collected across multiple sites.

**Supplementary Data 2**
Variance of gene expression explained by each of the covariates for uni-culture and village samples collected at Site 3 before and after cryopreservation.

**Supplementary Data 3**
hiPSC lines metrics.

**Supplementary Data 4**
Variance of gene expression explained by each of the covariates for fresh and cryopreserved samples across pseudotime.

**Supplementary Data 5**
Variance of gene expression explained by each of the covariates for villages collected during hiPSC village maintenance at passages one, four and eight.

**Supplementary Data 6**
Proportion of lines during cardiomyocyte differentiation.

**Supplementary Data 7**
Proportion of lines during multiple passages.

**Supplementary Data 8**
scCODA Metrics.
