# Supplementary material for: A village in a dish model system for population-scale hiPSC studies
Source: Nat Commun. 2023 Jun 9;14:3240. doi: 10.1038/s41467-023-38704-1 (PMC10256711; doi:10.1038/s41467-023-38704-1)
Supplement: Supplementary file 11 — Reporting Summary [file 41467_2023_38704_MOESM11_ESM.pdf]

nature portfolio

Corresponding author(s): Joseph Powell

Last updated by author(s): Feb 1, 2022

Reporting Summary

Nature Portfolio wishes to improve the reproducibility of the work that we publish. This form provides structure for consistency and transparency in reporting. For further information on Nature Portfolio policies, see our Editorial Policies and the Editorial Policy Checklist.

Statistics

For all statistical analyses, confirm that the following items are present in the figure legend, table legend, main text, or Methods section.

n/a	Confirmed	
☐	☒	The exact sample size (*n*) for each experimental group/condition, given as a discrete number and unit of measurement
☐	☒	A statement on whether measurements were taken from distinct samples or whether the same sample was measured repeatedly
☐	☒	The statistical test(s) used AND whether they are one- or two-sided<br>*Only common tests should be described solely by name; describe more complex techniques in the Methods section.*
☐	☒	A description of all covariates tested
☐	☒	A description of any assumptions or corrections, such as tests of normality and adjustment for multiple comparisons
☐	☒	A full description of the statistical parameters including central tendency (e.g. means) or other basic estimates (e.g. regression coefficient) AND variation (e.g. standard deviation) or associated estimates of uncertainty (e.g. confidence intervals)
☐	☒	For null hypothesis testing, the test statistic (e.g. *F*, *t*, *r*) with confidence intervals, effect sizes, degrees of freedom and *P* value noted<br>*Give P values as exact values whenever suitable.*
☐	☒	For Bayesian analysis, information on the choice of priors and Markov chain Monte Carlo settings
☒	☐	For hierarchical and complex designs, identification of the appropriate level for tests and full reporting of outcomes
☐	☒	Estimates of effect sizes (e.g. Cohen's *d*, Pearson's *r*), indicating how they were calculated

Our web collection on statistics for biologists contains articles on many of the points above.

Software and code

Policy information about availability of computer code

Data collection	No software was used in the collection of the data.
Data analysis	The following softwares were used through the course of the analysis of these data: 10x Genomics Cell Ranger Single Cell Software Suite (version 3.1.0), R (version 4.2.1), python (versions >= 3.6.8), scCODA (version 0.1.8), Seurat (version 4.0.0-4.0.5), Popscle (version 0.1-beta), scSplit (version 1.0.1), Souporcell (version 1.0), Vireo (version 0.4.2), DoubletDetection (version 3.0), scds (version 1.12.0), scrublet (version 1.0), scvelo (v0.2.3), pycombat (v0.3.0 and STAR (version 2.5.1b). Code for the scripts used for analysis are on Github (https://github.com/powellgenomicslab/iPSC_Village_Publication) and Zenodo (https://zenodo.org/record/7577941). Unprocessed and semi-processed data are available on GEO (GSE225282).

For manuscripts utilizing custom algorithms or software that are central to the research but not yet described in published literature, software must be made available to editors and reviewers. We strongly encourage code deposition in a community repository (e.g. GitHub). See the Nature Portfolio guidelines for submitting code & software for further information.

Data

Policy information about [availability of data](#)

All manuscripts must include a [data availability statement](#). This statement should provide the following information, where applicable:

- Accession codes, unique identifiers, or web links for publicly available datasets
- A description of any restrictions on data availability
- For clinical datasets or third party data, please ensure that the statement adheres to our [policy](#)

The raw and semi-processed data used for this publication is publicly available on GEO (GSE225282). The processed data is available on Zenodo (https://zenodo.org/record/7577941). Downloading instructions for GRCh38-1.2.0 (Ensembl release 84) are available from https://support.10xgenomics.com/single-cell-gene-expression/software/release-notes/build#grch38_1.2.0. The eQTL results from DeBoever et al were downloaded from the manuscript (Table S2: https://ars.els-cdn.com/content/image/1-s2.0-S1934590917300802-mmc3.xlsx).

Human research participants

Policy information about [studies involving human research participants and Sex and Gender in Research.](#)

Reporting on sex and gender	Sex-specific analyses were not performed. Y chromosome genes were interrogated for variance explained in the 3 hiPSC line village to confirm line-specific effects since Y chromosome genes should be expressed only by males.
Population characteristics	The hiPSC lines were derived from healthy individuals and were all from individuals of European ancestry.
Recruitment	No new patients were recruited for this study.
Ethics oversight	The research carried out in this study was in accordance with the Declaration of Helsinki and approved by the Human Research Ethics committees of the University of Melbourne (1545394), the Garvan Institute of Medical Research (ETH01307) and the University of Queensland (2015001434).

Note that full information on the approval of the study protocol must also be provided in the manuscript.

Field-specific reporting

Please select the one below that is the best fit for your research. If you are not sure, read the appropriate sections before making your selection.

☒ Life sciences ☐ Behavioural & social sciences ☐ Ecological, evolutionary & environmental sciences

For a reference copy of the document with all sections, see [nature.com/documents/nr-reporting-summary-flat.pdf](#)

Life sciences study design

All studies must disclose on these points even when the disclosure is negative.

Sample size	Previous studies have demonstrated that 1,000 cells per individual were sufficient to identify individual-specific effects (Yazar et al, Science, 2022). Since our samples were less heterogeneous than the samples in Yazar et al, we rationalized that ~1,000 cells per line would be sufficient to study iPSC line variation. Therefore, we aimed to capture ~1,500 single cells per hiPSC line in each sample for the 3-line hiPSC village and ~1,000 single cells per hiPSC line in each sample for the 18-line hiPSC lines.
Data exclusions	Some single cells were removed because they were classified as multiplets (single cell droplets containing more than one cell) because they would add noise to the analysis. Additional cells were removed it they had high mitochondrial percent or contained a low number of detected genes.
Replication	Triplicates of each experimental condition were used for the 3-line hiPSC village and thousands of cells per sample were collected - acting as additional replicates. The triplicates at each site were highly consistent. Replicates were not produced for the cardiac differentiation village or multi-passage village since the triplicates in the three-line village were highly consistent suggesting that replicates were not needed and that the thousands of cells captured could be used as replicates. Thousands of cells were captured per sample and different samples were captured over multiple days or passages to interrogate long-term trends. All data generated for this study were analyzed and no replications or experiments were left out. Growth rate experiments were carried out in triplicate and all data were included in the analysis.
Randomization	No randomization was required for this study as each hiPSC line was used at each site and condition to estimate the variance explained by different covariates.
Blinding	No blinding was used in the hiPSC village single cell captures. The different hiPSC lines, sites and replicates were captured at the same time to reduce capture biases. The results of the proportions of each hiPSC line from the single cell experiments was blinded to the experimenters carrying out the growth rate experiments. No other experiments were performed.

Reporting for specific materials, systems and methods

We require information from authors about some types of materials, experimental systems and methods used in many studies. Here, indicate whether each material, system or method listed is relevant to your study. If you are not sure if a list item applies to your research, read the appropriate section before selecting a response.

Materials & experimental systems

n/a	Involved in the study
☒	☐ Antibodies
☐	☒ Eukaryotic cell lines
☒	☐ Palaeontology and archaeology
☒	☐ Animals and other organisms
☒	☐ Clinical data
☒	☐ Dual use research of concern

Methods

n/a	Involved in the study
☒	☐ ChIP-seq
☐	☒ Flow cytometry
☒	☐ MRI-based neuroimaging

Eukaryotic cell lines

Policy information about cell lines and Sex and Gender in Research

Cell line source(s)	The hiPSC lines were generated by Alice Pebay's lab and donated as part of the collaboration.
Authentication	The cell line was SNP genotyped following production of the hiPSC lines and matched to genotype data from the original fibroblast samples donated by each individual.
Mycoplasma contamination	All cell lines tested negative for mycoplasma.
Commonly misidentified lines (See ICLAC register)	None of the cell lines used in this study are commercially available. Therefore, no commonly misidentified cell lines were used.

Flow Cytometry

Plots

Confirm that:

☒ The axis labels state the marker and fluorochrome used (e.g. CD4-FITC).

☒ The axis scales are clearly visible. Include numbers along axes only for bottom left plot of group (a 'group' is an analysis of identical markers).

☒ All plots are contour plots with outliers or pseudocolor plots.

☒ A numerical value for number of cells or percentage (with statistics) is provided.

Methodology

Sample preparation	One million cells from each replicate at each site were centrifuged at 300g for three minutes and the supernatant was discarded. The cell pellets were resuspended in 100µL cold Fluorescence-Activated Cell Sorting (FACS) buffer (phosphate buffered saline [PBS] with two percent fetal bovine serum [FBS]). Then, 2µL of Totalseq-A hashing antibody was added and the cells were gently pipetted to mix before incubating for 20 minutes on ice. Cells were then washed twice with FACS buffer by centrifuging at 300g for 5 minutes, discarding the supernatant and resuspending the cell pellet in 100µL cold FACS buffer. Cells were briefly stained with 4',6-diamidino-2-phenylindole (DAPI) before using flow cytometry (BD FACS Aria, 100um nozzle, 4-way purity mode, temperature controlled) to sort and capture live single cells.
Instrument	BD FACS Aria, 100um nozzle, 4-way purity mode, temperature controlled
Software	Collection of Data: FACS Diva v8.01<br>Analysis of flow cytometry data: Flowjo v10.6.1
Cell population abundance	Cells were sorted for live cells. Captured cells were 42% of starting material and 82% of the single cell population
Gating strategy	Cells were selected using forward and side area scatter. The single cells were determined from a combination of forward scatter height, width and area and side scatter of height, width and area ratios. DAPI was used to stain samples and perform live cell gating using V450 area vs B530 area.

☒ Tick this box to confirm that a figure exemplifying the gating strategy is provided in the Supplementary Information.
